# Long-term adaptation of lymphoma cell lines to hypoxia is mediated by diverse molecular mechanisms that are targetable with specific inhibitors

**DOI:** 10.1038/s41420-025-02341-y

**Published:** 2025-02-18

**Authors:** Lenka Daumova, Dmitry Manakov, Jiri Petrak, Dana Sovilj, Matej Behounek, Ladislav Andera, Ondrej Vit, Olga Souckova, Ondrej Havranek, Alex Dolnikova, Nicol Renesova, Liliana Tuskova, Lucie Winkowska, Nardjas Bettazova, Kristyna Kupcova, Marie Hubalek Kalbacova, Miriama Sikorova, Marek Trneny, Pavel Klener

**Affiliations:** 1 Institute of Pathological Physiology, First Faculty of Medicine, Charles University, Prague, Czech Republic; 2 BIOCEV Biotechnology and Biomedicine Centre, First Faculty of Medicine, Charles University, Vestec, Czech Republic; 3 Institute of Biotechnology, Czech Academy of Sciences / BIOCEV, Vestec, Czech Republic; 4 Institute of Molecular Genetics, Czech Academy of Sciences, Prague, Czech Republic; 5 OMICS Mass Spectrometry Core Facility, Biology Departments, BIOCEV, Faculty of Science, Charles University, Vestec, Czech Republic; 6 First Department of Medicine- Hematology, University General Hospital Prague and First Faculty of Medicine, Charles University, Prague, Czech Republic; 7 CLIP, Department of Paediatric Haematology and Oncology, Second Faculty of Medicine, Charles University and University Hospital Motol, Prague, Czech Republic; 8 Department of Medical Genetics, Third Faculty of Medicine, Charles University, Prague, Czech Republic

**Keywords:** B-cell lymphoma, Cell growth

## Abstract

A large body of evidence suggests that hypoxia drives aggressive molecular features of malignant cells irrespective of cancer type. Non-Hodgkin lymphomas (NHL) are the most common hematologic malignancies characterized by frequent involvement of diverse hypoxic microenvironments. We studied the impact of long-term deep hypoxia (1% O2) on the biology of lymphoma cells. Only 2 out of 6 tested cell lines (Ramos, and HBL2) survived ≥ 4 weeks under hypoxia. The hypoxia-adapted (HA)b Ramos and HBL2 cells had a decreased proliferation rate accompanied by significant suppression of both oxidative phosphorylation and glycolytic pathways. Transcriptome and proteome analyses revealed marked downregulation of genes and proteins of the mitochondrial respiration complexes I and IV, and mitochondrial ribosomal proteins. Despite the observed suppression of glycolysis, the proteome analysis of both HA cell lines showed upregulation of several proteins involved in the regulation of glucose utilization including the active catalytic component of prolyl-4-hydroxylase P4HA1, an important druggable oncogene. HA cell lines demonstrated increased transcription of key regulators of auto-/mitophagy, e.g., neuritin, BCL2 interacting protein 3 (BNIP3), BNIP3-like protein, and BNIP3 pseudogene. Adaptation to hypoxia was further associated with deregulation of apoptosis, namely upregulation of BCL2L1/BCL-XL, overexpression of BCL2L11/BIM, increased binding of BIM to BCL-XL, and significantly increased sensitivity of both HA cell lines to A1155463, a BCL-XL inhibitor. Finally, in both HA cell lines AKT kinase was hyperphosphorylated and the cells showed increased sensitivity to copanlisib, a pan-PI3K inhibitor. In conclusion, our data report on several shared mechanisms of lymphoma cell adaptation to long-term hypoxia including: 1. Upregulation of proteins responsible for glucose utilization, 2. Degradation of mitochondrial proteins for potential mitochondrial recycling (by mitophagy), and 3. Increased dependence on BCL-XL and PI3K-AKT signaling for survival. In translation, inhibition of glycolysis, BCL-XL, or PI3K-AKT cascade may result in targeted elimination of HA lymphoma cells.

## Introduction

Non-Hodgkin lymphomas (NHL) represent the most common hematologic malignancies [1, 2]. NHLs are typically characterized by widespread disease associated with infiltration of lymph nodes and secondary lymphoid organs (e.g., the spleen), as well as many extra-nodal sites, including bone marrow (BM), gastrointestinal tract, central nervous system (CNS), peripheral blood, or other organs and tissues. Compared to the arterial blood with high oxygen levels (average 13% O_2_), lymph nodes, spleen, or BM are characterized by extravascular hypoxic gradients. For example, BM and spleen have average 1.3% and 2.3% O_2_, respectively [3]. Several other lymphoma microenvironments, e.g., malignant effusions, CNS, or large lymphoma masses with central necroses are also characterized by hypoxic gradients with very low nadirs of oxygen levels [4–8]. It is important to underline that in these hypoxic niches, lymphoma cells are exposed to low oxygen levels long-term (i.e., weeks to months).

Large body of evidence suggests that hypoxia drives aggressive molecular features irrespective of cancer types [3, 9–13]. Stabilization of hypoxia-inducible transcription factor (HIF1α) belongs to key mechanisms of hypoxia-triggered angiogenesis, metabolic adaptation, metastasizing, or drug resistance [3, 14, 15]. In chronic lymphocytic leukemia, HIF1α increased colonization of BM and spleen via transactivation of genes involved in chemotaxis and interaction of malignant B-cells with these microenvironments [14]. In addition to hypoxia, HIF1α can be upregulated because of activation of oncogenes or inactivation of tumor suppressors. It was demonstrated that HIF1α is required for survival of leukemia stem cells and that HIF1α target genes are specifically upregulated in *TP53*-mutated versus *TP53*-wild-type leukemia cells [16–18]. Consequently, HIF1α-induced druggable targets may be utilized for effective eradication of hypoxia exposed, HIF-reprogrammed leukemic cells [19, 20].

The transcriptional or proteomic changes associated with short term hypoxia (24–72 h) was repeatedly studied [21]. In contrast, the impact of long-term deep hypoxia (1% O_2_, 5% CO_2_) on the biology of lymphoma cells, as well as the potential role of long-term deep hypoxia-induced adaptive changes in lymphoma clonal evolution, treatment resistance, and disease relapses remain largely unexplored.

In this study we derived lymphoma cell lines adapted to long term (≥4 weeks) deep (1% O_2_, 5% CO_2_) hypoxia and implemented a complex transcriptional, proteomic, metabolic, and functional analysis of hypoxia-adapted (HA) lymphoma cells.

## Results

### NHL cell lines show different responses to long-term hypoxia

**Fig. 1:**
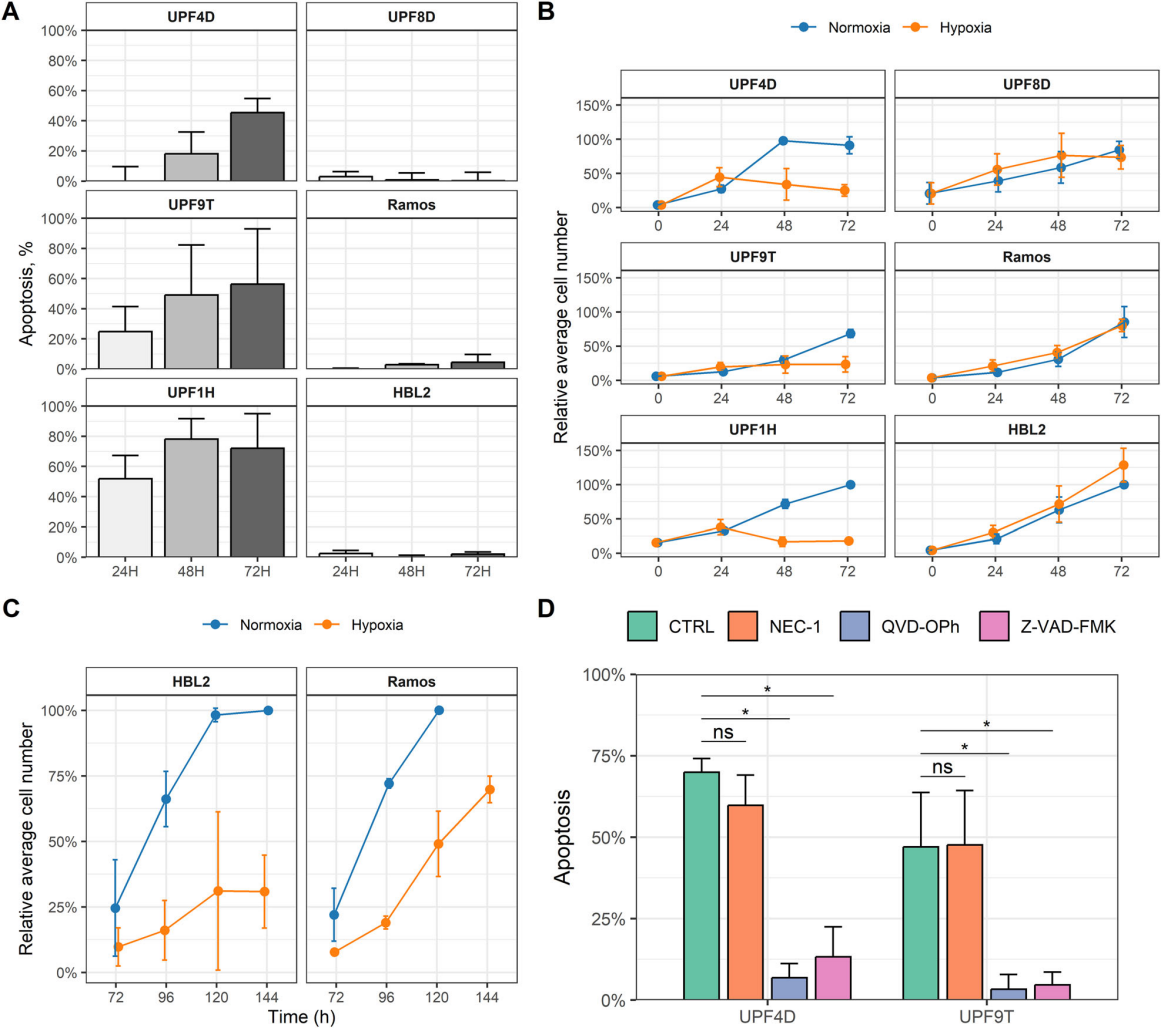
Different sensitivity of lymphoma cells to hypoxia. **A** Percentage of apoptotic cells 24, 48, and 72 h after placement of the cells into hypoxia (1% O_2_); (**B**) proliferation rate of lymphoma cells cultured under standard normoxic (blue) and hypoxic (1% O_2_, orange) conditions during the first three days from the placement of the cells into hypoxia (0, 24, 48, and 72 h). At 0 h, 10 thousand cells per well were placed into 96-well plate and proliferation rate was measured for 3 consecutive days in triplicates (blue= normoxic conditions, orange= hypoxic conditions); (**C**) proliferation rate of lymphoma cells cultured under standard normoxic and hypoxic (1% O_2_) conditions from 72 until 144 h of the placement of the cells into hypoxia. After 72 h of the culture under hypoxia, 10 thousand cells per well were placed into a new 96-well plate supplemented with fresh medium and proliferation rate was measured for another 3 consecutive days in triplicates (blue= normoxic conditions, orange= hypoxic conditions); (**D**) hypoxia-induced cell death in hypoxia-sensitive lymphoma cell lines (UPF4D, UPF9T) can be blocked by Z-VAD-FMK (20 μM), a pan-caspase inhibitor or Q-VD-OPh (20 μM), a broad spectrum caspase inhibitor, but not by the inhibitor of necroptosis necrostatin 1 (NEC-1, 20 μM). *Y* axis shows percentage of Annexin V-positive cells. In all cases, results represent means ± standard deviations (SD) of at least two independently implemented experiments.

First, we analyzed apoptosis and proliferation rates of different lymphoma cell lines exposed to short-term deep hypoxia (1% O_2_, referred to as “hypoxia” throughout this manuscript). Six lymphoma cell lines including diffuse large B-cell lymphoma (DLBCL, UPF4D, UPF8D), Burkitt lymphoma (UPF9T, Ramos), and mantle cell lymphoma (MCL, UPF1H, HBL2) demonstrated different responses to 3-day culture under uninterrupted hypoxia. While three out of six tested cell lines (UPF4D, UPF9T, UPF1H) were sensitive to hypoxia (≥50% apoptotic cells and significantly decreased proliferation rate after 72 h of culture in hypoxia), the other three lymphoma cell lines (UPF8D, Ramos, and HBL2) appeared (seemingly) resistant to short-term hypoxia in terms of apoptosis and proliferation rates (as compared to normoxia, Fig. 1A, B). However, only two of them, namely Ramos and HBL2 survived long-term culture (≥4 weeks) under uninterrupted deep hypoxia (1% O_2_) yielding so called hypoxia-adapted (HA) cells. UPF8D cells did not survive long-term culture under deep hypoxia.

Although there was no obvious inhibition of Ramos and HBL2 cell proliferation during the first 3 days under hypoxia, their proliferation rate significantly declined starting from day 4 under uninterrupted hypoxia (Fig. 1C). The proliferation rate of both HA Ramos and HBL2 cells (cultured for ≥ 4 weeks under uninterrupted hypoxia) was significantly slower compared to the proliferation rate of the original Ramos and HBL2 cell lines cultured in parallel under standard normoxic tissue culture conditions (Supplementary Fig. 2).

Finally, using lymphoma cell lines highly sensitive to hypoxia (UPF4D, UPF9T) we demonstrated that the observed cytotoxic effect of hypoxia was effectively blocked by inhibitors of caspases (Z-VAD-FMK, QVD-OPh), suggesting that hypoxia triggers caspase-dependent apoptosis (Fig. 1D).

### HA lymphoma cell lines have significantly reduced oxidative phosphorylation and glycolytic pathways

**Fig. 2:**
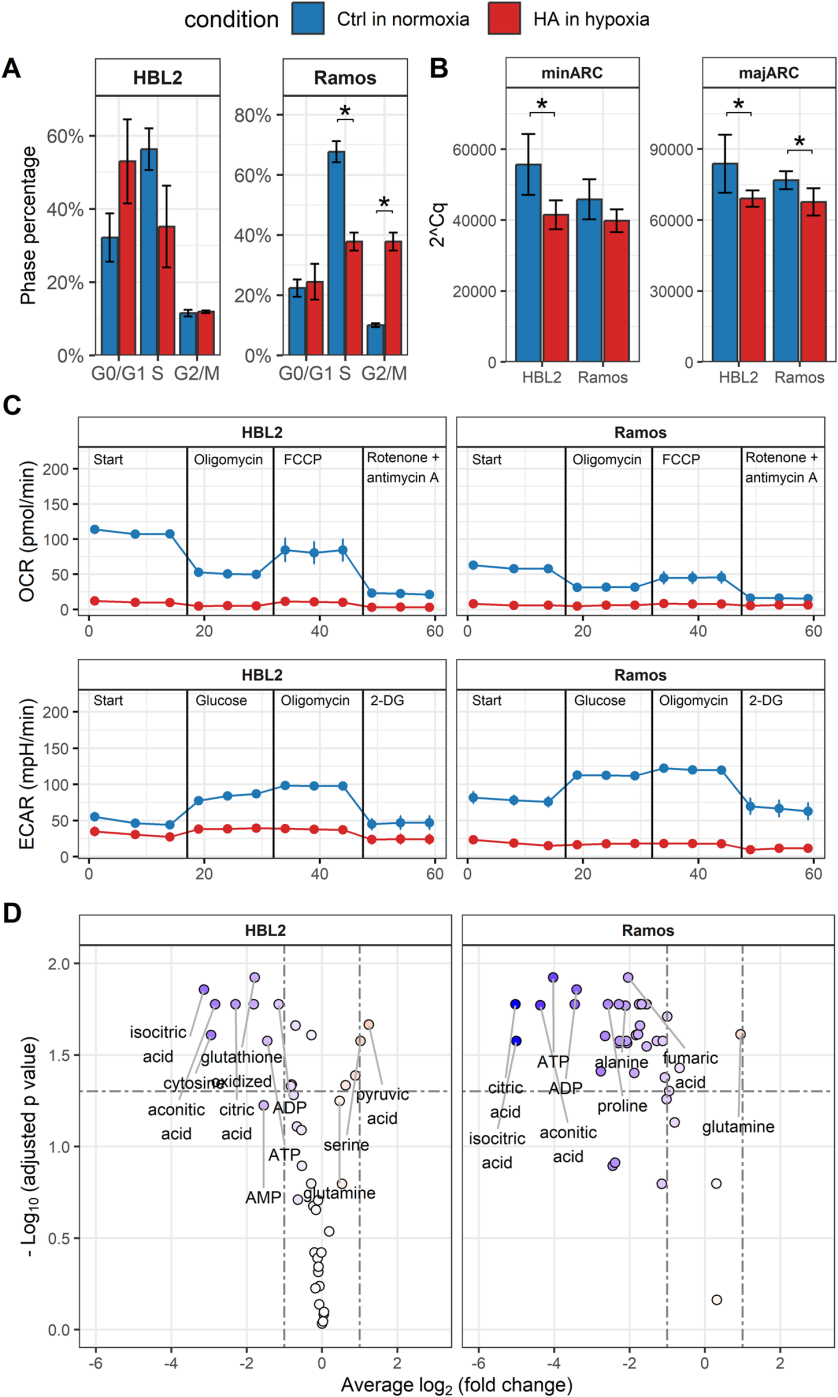
Decreased metabolic activity of HA lymphoma cells. **A** Cell cycle analysis of hypoxia-adapted (HA) lymphoma cells cultured in uninterrupted hypoxia (red, 1% O_2_) compared to the original lymphoma cells cultured under standard normoxic tissue culture conditions (blue); results represent means ± standard deviations (SD) of two independently implemented experiments. **B** Mitochondrial DNA content quantified using q(uantitative)PCR in HA lymphoma cells cultured under uninterrupted hypoxia (red, 1% O_2_) compared to the original lymphoma cells cultured under standard normoxic tissue culture conditions (blue, * - *p* < 0.05). minARC – minor mitochondrial DNA arc, majARC – major mitochondrial DNA arc; (**C**) analysis of oxygen consumption rate (OCR) and extracellular acidification rate (ECAR) of HA lymphoma cells cultured in uninterrupted hypoxia (red, 1% O_2_) compared to the original lymphoma cell lines cultured under standard normoxic tissue culture conditions (blue) using Seahorse XFe 96 analyzer; (**D**) volcano plots of relative metabolite abundance in HA lymphoma cells cultured in uninterrupted hypoxia compared to the respective cell lines (cultured under standard normoxic tissue culture conditions). Values are expressed as log2 (fold change). Only the metabolites which were over the detection limit in both conditions are plotted.

As mentioned above, both Ramos and HBL2 HA cells (cultured for ≥4 weeks under uninterrupted hypoxia) demonstrated significantly slower proliferation rate compared to the original cell lines cultured under normoxic conditions (Supplementary Fig. 2). The slower proliferation rate was associated with a decreased fraction of the HA cells in the S phase of the cell cycle and an increased fraction of the HA cells in the G2/M phase in case of Ramos (but not HBL2) cells (Fig. 2A). The content of mitochondrial DNA was decreased in both HA cells in comparison to their normoxic controls suggesting long-term hypoxia-induced mitochondrial deregulation (Fig. 2B). Indeed, analysis of oxygen consumption rate (OCR) and extracellular acidification rate (ECAR) by SeaHorse XFe 96 analyzer revealed significant downregulation of both oxidative phosphorylation and glycolysis in both HA cell lines (Fig. 2C). In addition, the metabolomics analysis also confirmed significant downregulation of most of the measured metabolites in both HA cell populations compared to the respective normoxic controls. Specifically, reduction in oxidative metabolism was documented by significantly reduced content of ATP, and ADP. Metabolites of the citric acid cycle (citrate, isocitrate, aconitate, succinate, fumarate, malate) were decreased in both HA cell lines, as well as the levels of oxidized and reduced glutathione. In contrast, glutamine levels were increased in the HA cells, which may suggest increased utilization of glutamine as an alternative substrate during the lymphoma cell proliferation under uninterrupted long-term hypoxic conditions. HA Ramos cells differed from HA HBL2 cells by pronounced and wide depletion of amino acids including asparagine (Asn), proline (Pro), threonine (Thr), tryptophane (Trp), valine (Val), alanine (Ala), serine (Ser), tyrosine (Tyr), leucine (Leu), glycine (Gly), lysine (Lys), isoleucine (Ileu), and histidine (His) (Fig. 2D, Supplementary Tables 3–4). The complete list of the analyzed metabolites can be downloaded from 10.5281/zenodo.10993246.

### HA cells are significantly more sensitive to inhibitors of glycolysis, BCL-XL, and PI3K-AKT cascade

**Fig. 3:**
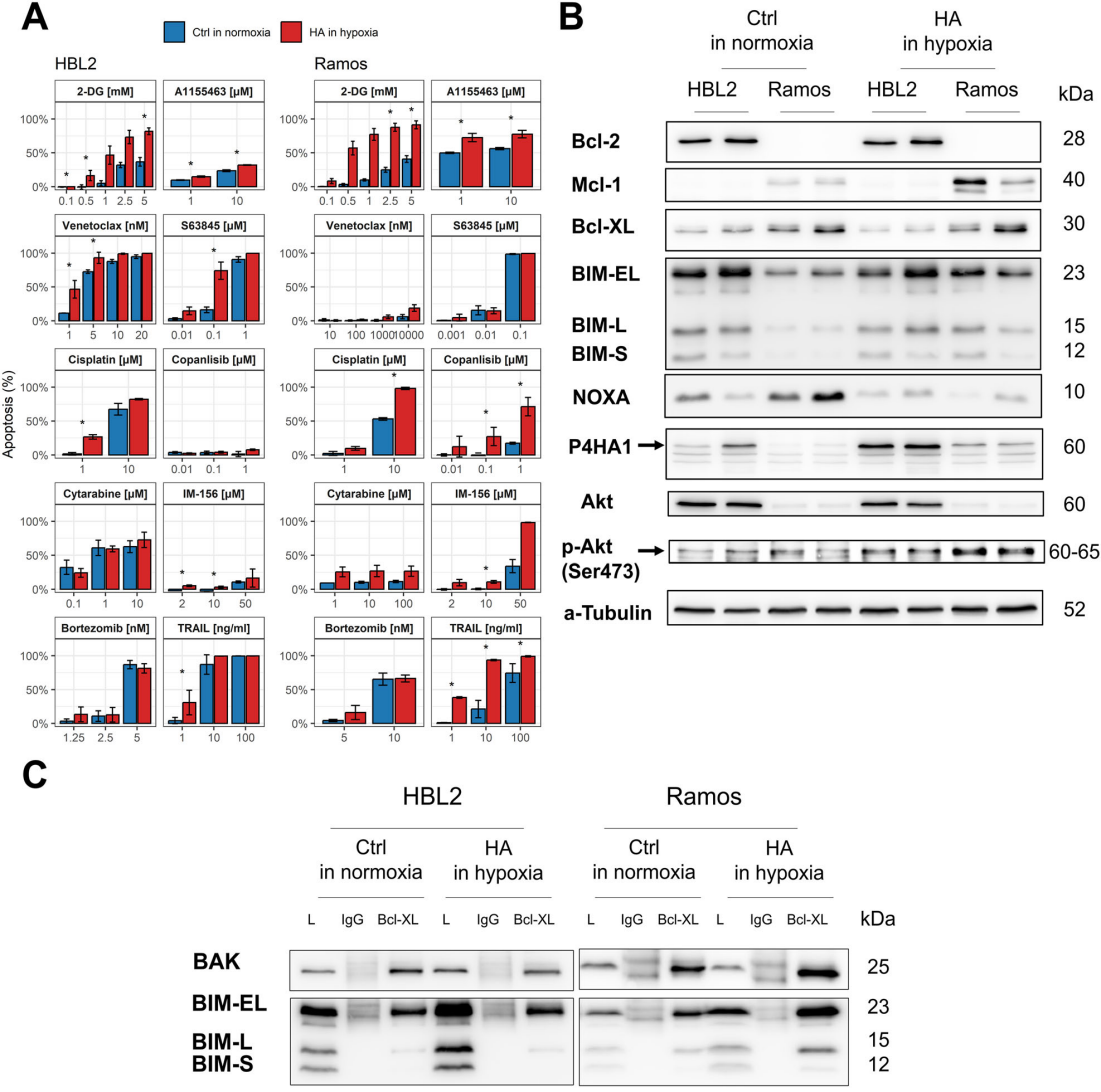
Changed sensitivity of HA lymphoma cells (cultured in hypoxia) to the selected anti-cancer agents. **A** Apoptosis was determined 24 h after exposure of the original lymphoma cell lines cultured under standard normoxic tissue culture conditions (blue) and HA cells cultured in uninterrupted hypoxia (red), 2-DG = 2-deoxy-glucose (inhibitor of glycolysis), IM-156 = inhibitor of oxidative phosphorylation, venetoclax, A1155463, and S63845 = inhibitor of BCL2, BCL-XL, and MCL1, respectively, TRAIL = Tumor necrosis factor alpha-related apoptosis-inducing ligand, copanlisib = pan-AKT inhibitor; cisplatin, cytarabine = genotoxic cytostatics; bortezomib = proteasome inhibitor; results represent means ± standard deviations (SD) of three independently implemented experiments; (**B**) western blot analysis of biological duplicates of HA cells (cultured in uninterrupted hypoxia) compared to the original lymphoma cell lines (cultured in standard normoxic tissue culture conditions); (**C**) immunoprecipitation (IP) of the HA lymphoma cells (cultured in uninterrupted hypoxia) compared to the original lymphoma cell lines (cultured in standard normoxic tissue culture conditions) with anti-BCL-XL antibody and subsequent detection of proapoptotic BIM and BAK bound on BCL-XL; increased amounts of proapoptotic BIM could be detected in total lysates of both HA HBL2 and HA Ramos cells (compared to the normoxic controls); increased amount of BIM was detected bound on BCL-XL; L= total lysates, IgG stands for lysates immunoprecipitated with polyclonal rabbit IgG; BCL-XL stands for lysates immunoprecipitated with anti-BCL-XL antibody; BIM-EL/L/S = extra-large / large / small BIM isoforms. Increased amount of proapoptotic effector BAK was detected bound on BCL-XL in HA Ramos cells. Proapoptotic BAX was not detected bound on BCL-XL in either tested cell line.

We have demonstrated that adaptation to long-term uninterrupted hypoxia (1% O_2_) led to significant changes in the proliferation rate, energy-metabolic pathways, and metabolome. We thus asked if these changes may have impact on sensitivity of HA lymphoma cells to anti-cancer agents. Therefore, HA HBL2 and HA Ramos cells (cultured continuously for ≥ 4 weeks under uninterrupted hypoxic conditions) and the original HBL2 and Ramos cell lines (cultured continuously under standard normoxic tissue culture conditions) were exposed to a battery of anti-cancer agents. In most cases, the HA cells were more sensitive to the tested anti-cancer agents than their normoxic counterparts. However, the biggest differences were observed in the markedly increased sensitivity of HA cells to 2-deoxy-glucose (2-DG), an inhibitor of glycolysis and A1155463, an inhibitor of anti-apoptotic B-cell lymphoma 2 (BCL2)-like 1 (BCL2L1/BCL-XL) protein (Fig. 3A). The observed increased sensitivity of HA cells to the inhibitor of BCL-XL A1155463 (and in case of HA HBL2 also to the other two tested mitochondrial targeting agents, i.e., venetoclax, a BCL2 inhibitor and S63845, a MCL1 inhibitor) suggested changed expression of anti- and pro-apoptotic BCL2 family proteins, and/or increased priming of HA cells for death. Indeed, western blot analysis confirmed upregulation of pro-apoptotic activator BCL2-like 11 (BCL2L11/BIM), and downregulation of pro-apoptotic sensitizer phorbol-12-myristate-13-acetate-induced protein 1 (PMAIP1/NOXA) in both HA lymphoma cell lines (compared to the respective cell lines cultured under normoxia) (Fig. 3B). In addition, immunoprecipitation experiments confirmed increased binding of BIM to BCL-XL in both HA cell lines (Fig. 3C). By western blot analysis, the AKT kinase was hyperphosphorylated in both HA lymphoma cell lines suggesting adaptive overactivation of the PI3K/AKT cascade in response to long-term deep uninterrupted hypoxia (Fig. 3B).

### Transcriptome analysis

**Fig. 4:**
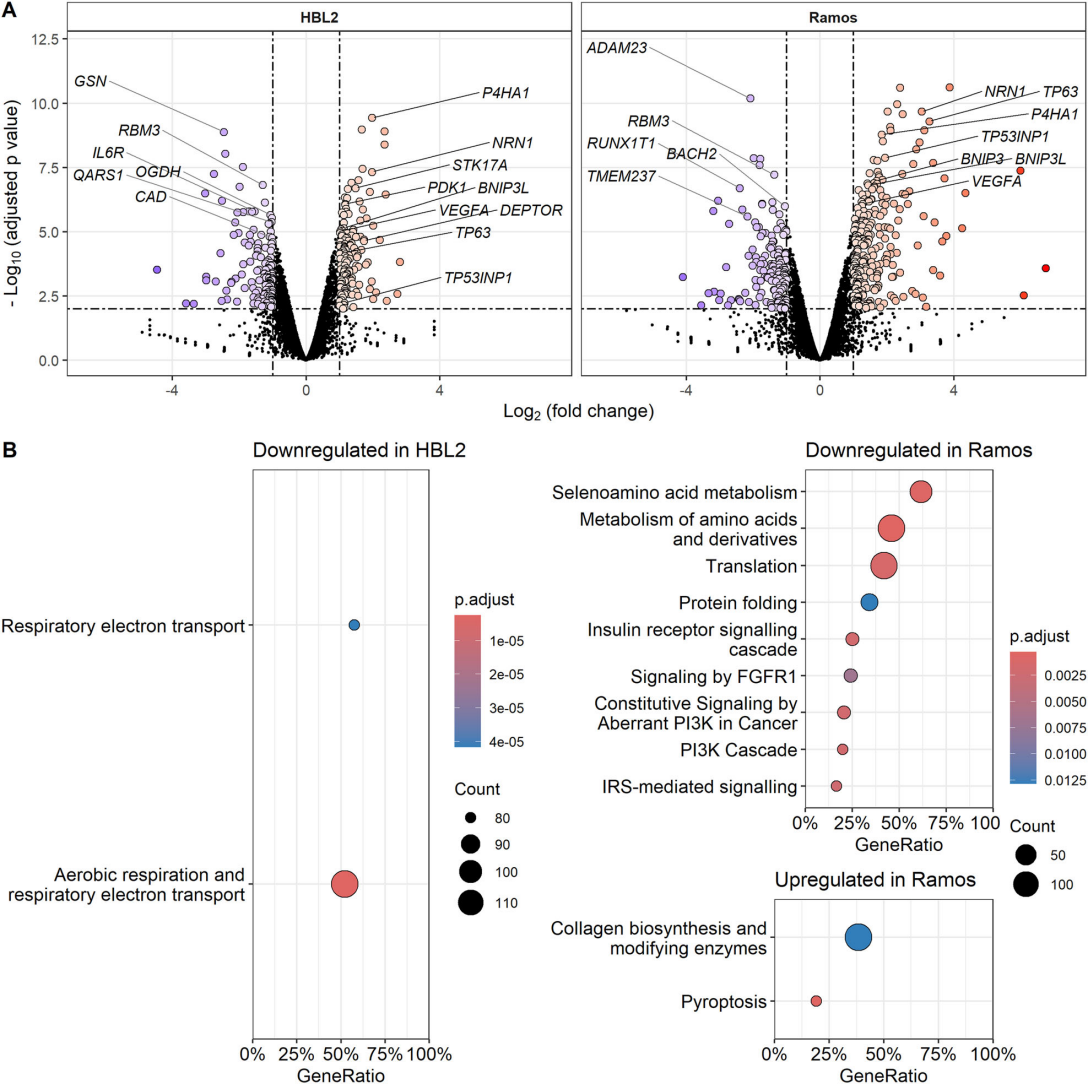
Transcriptome analysis of HA HBL2 and HA Ramos cells (cultured in hypoxia) compared to their respective counterparts cultured under normoxia. **A** Volcano plots showing fold change and significance of detected protein coding transcripts. **B** Gene set enrichment analysis dot plots. GeneRatio—proportion of detected genes to total number of genes in a pathway. The transcriptome analysis was carried out using biological triplicates of the HA cells compared to their normoxic controls.

**Fig. 5:**
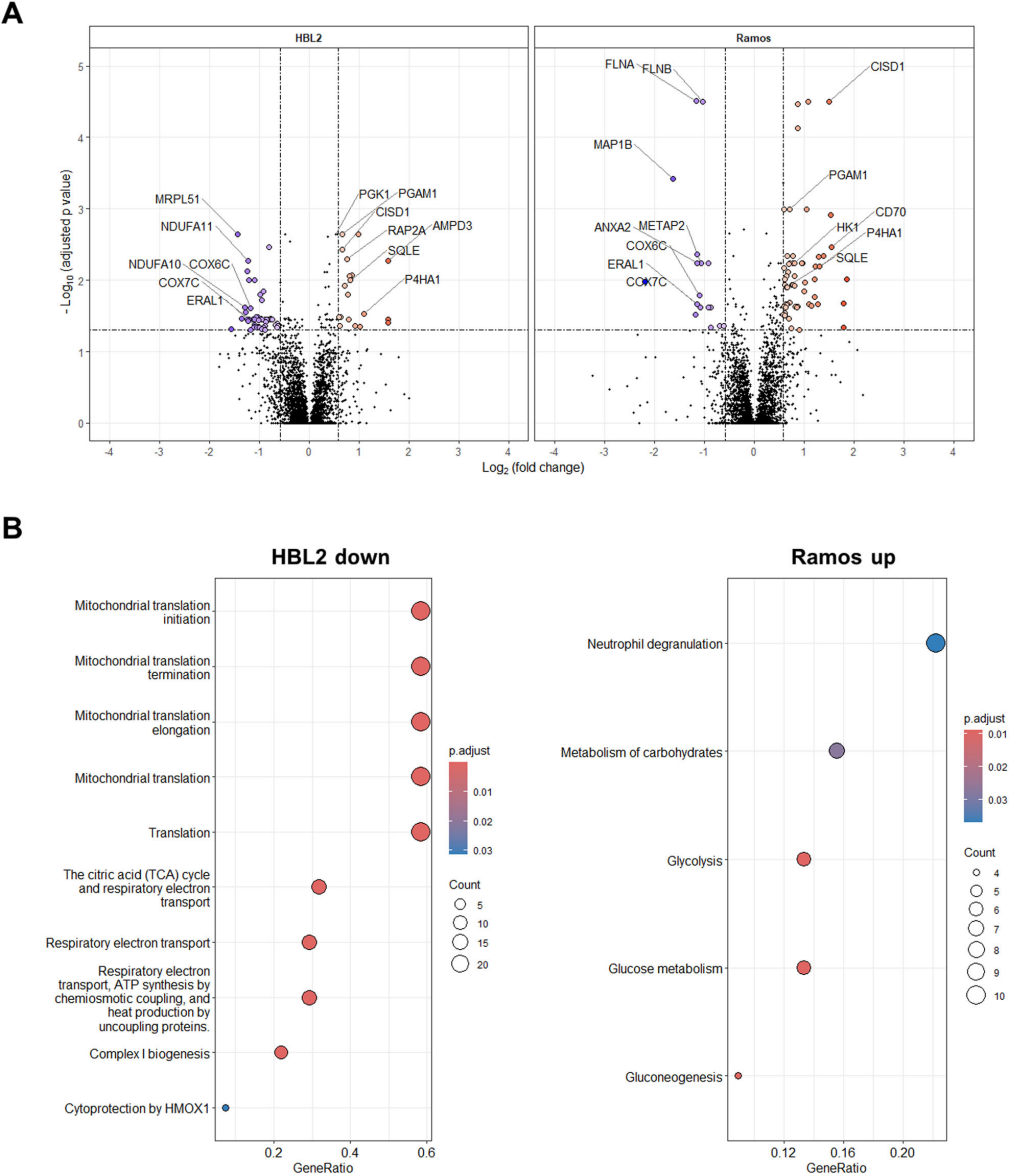
Proteome analysis of HA HBL2 and Ramos cells compared to their respective counterparts cultured under normoxia. **A** Volcano plots show fold change and significance of detected proteins. **B** Gene set enrichment analysis dot plots. GeneRatio—proportion of detected genes to total number of genes in a pathway. The proteome analysis was carried out using biological triplicates of the HA cells compared to their normoxic controls.

To gain deeper insight into molecular changes associated with adaptation of lymphoma cell lines to long-term uninterrupted hypoxia, both HA cell lines (cultured continuously for ≥ 4 weeks under 1% O_2_ hypoxia) were subject to transcriptome and proteome analyses compared to their respective parental counterparts cultured under standard normoxic tissue culture conditions (Figs. 4, 5). RNA sequencing analysis identified 13,466 total transcripts, of which 242 were upregulated and 174 were downregulated in HA HBL2 cells and 390 were upregulated and 194 were downregulated in HA Ramos cells (both in comparison to the respective normoxic controls). Statistical significance thresholds were set at fold change 2, *p* value < 0.05 and FDR < 0.25. The complete list of differentially expressed genes can be downloaded from 10.5281/zenodo.10993246. Differentially expressed genes with >2-fold change (FC) and *p* value < 1E–06 are listed in Supplementary Tables 5–8.

Vascular endothelial growth factor A (*VEGFA*), a known HIF1α target, was significantly upregulated in HA Ramos (FC = 2.4, *p* = 8.36^E–07^), as well as in HA HBL2 cells (FC = 2.11, *p* = 9.09^E–06^). In addition, differentially expressed transcripts detected in both HA HBL2 and HA Ramos cells comprised prolyl 4-hydroxylase subunit alpha-1 (*P4HA1*), involved in collage biosynthesis, glycolysis, and cancer progression, regulators of autophagy/mitophagy neuritin (*NRN1*), BCL2 interacting protein 3 like (*BNIP3L*), and regulators of DNA damage response tumor protein p63 (*TP63*), tumor protein p53 inducible nuclear protein 1 (*TP53INP1*), and serine/threonine kinase 17a (*STK17A*). In HBL2, BNIP3 and BNIP3 pseudogene 1 (*BNIP3P1*) were upregulated together with *BNIP3L*. Transcripts downregulated in both HA HBL2, and Ramos cells comprised RNA binding motif protein 3 (*RBM3*), a known suppressor of *TP53* protein translation.

Gene set enrichment analysis (GSEA) using Reactome pathways identified 2 significantly downregulated pathways in HA HBL2 and 42 downregulated and 7 upregulated pathways in HA Ramos. Pathways downregulated in HA HBL2 cells included aerobic respiration and respiratory electron transport (normalized enrichment score [NES] = -2.07, adjusted *p* value = 2.48E–06) and respiratory electron transport (NES = –2.16, adjusted *p* value = 4.16E–05). Pathways downregulated in HA Ramos cells comprised metabolism of amino acids and derivatives (NES = –1.82, adjusted p value = 0.00029), translation (NES = –1.71, adjusted *p* value = 0.0013), and protein folding (NES = -1.85, adjusted *p* value = 0.012), which was in concordance with the previous metabolomics analysis that had demonstrated depletion of amino acids. In addition, pathways downregulated in HA Ramos cells included insulin receptor signaling, fibroblast growths factor receptor 1 (FGFR1) signaling, and PI3K signaling. Pathways upregulated in HA Ramos cells included collagen biosynthesis and formation, and pyroptosis (Fig. 4B, the complete list of differentially expressed pathways can be downloaded from 10.5281/zenodo.10993246).

### Proteomic analysis

To better understand the molecular changes associated with long-term adaptation of lymphoma cells to uninterrupted deep hypoxia, both HA cell lines (cultured continuously for ≥4 weeks under hypoxia) were also subject to proteome analyses compared to their respective parental counterparts cultured under standard normoxic tissue culture conditions. Multiplexed proteomic analysis employing labeling with TMT stable isotope tags provided identification of over 5 100 proteins identified with at least two unique peptides. Of those, 4 443 and 4435 provided quantitative and statistical data in HBL2 and Ramos cells, respectively. The complete list of differentially expressed genes can be downloaded from 10.5281/zenodo.10993246. The mass spectrometry proteomics data have been deposited to the ProteomeXchange Consortium via the PRIDE partner repository with the dataset identifier PXD050227.

The adaptation to hypoxia in HBL2 cells resulted in differential expression (>1.5 FC, adj. *p* value < 0.05) of 68 proteins. Of those 20 proteins were upregulated, and 48 downregulated. Hypoxia adaptation in Ramos cells resulted in altered expression of 76 proteins. Of those, 59 proteins were upregulated and 17 downregulated (Fig. 5, Supplementary Tables 9–12). Proteins significantly (FC > 1.5) upregulated in both HA HBL2, and HA Ramos cells included prolyl 4-hydroxylase subunit alpha-1 (P4HA1), AMP deaminase 3 (AMPD3), phosphoglycerate mutase 1 (PGAM1), squalene monooxygenase (SQLE), or CDGSH iron-sulfur domain-containing protein (CISD1). Proteins downregulated in both HA HBL2, and HA Ramos cells included cytochrome c oxidase subunit 6 C and 7 C (COX6C, COX7C), or mitochondrial GTPase Era (ERAL1).

Importantly, HBL2 cell line adaptation to long-term hypoxia was associated with massive downregulation of mitochondrial proteins. In fact, only 6 out of the 48 proteins downregulated in HA HBL2 cells were from other compartments than the mitochondria. Specifically, we observed decreased levels of mitochondrial ribosome subunits and proteins of ribosome assembly (24 + 4 proteins, respectively) and components of mitochondrial ETC Complex I (9 proteins) and Complex IV (2 proteins) along with 3 additional mitochondrial proteins including isocitrate dehydrogenase subunit IDH3B. Proteins upregulated in HA HBL2 cells comprised enzymes involved in utilization of glucose including enolase 2 (ENO2), phosphoglycerate mutase 1 (PGAM1), P4HA1, regulators of ferroptosis CDGSH iron sulfur domain 1 (CISD1), squalene epoxidase (SQLE), and several important oncogenes including tyrosine kinase LCK, high mobility group nucleosome binding domain 5 (HMGN5), or dihydropyrimidinase like 2 (DPYSL2).

In Ramos cells, adaptation to hypoxia led to metabolic reprogramming toward utilization of glucose exemplified by preferential upregulation of proteins involved in glucose metabolism including enolase 1 (ENOA), aldolase, fructose-bisphosphate C (ALDOC), pyruvate kinase M1/2 (PKM), phosphoglycerate mutase 1 (PGAM1), hexokinase 1 (HK1), triosephosphate isomerase 1 (TPI), or the glucose transporters GLUT1 (SLC2A1), and GLUT5 (SLC2A5). Similarly to HA HBL2, markers and regulators of ferroptosis, e.g., ferritin light chain (FTL), CISD1, and CISD2 were also upregulated in HA Ramos cells, as well as prolyl 4-hydroxylase subunit alpha-1 (P4HA1). P4HA1 was one of a few molecules that were significantly upregulated at the mRNA as well as at the protein levels in both HA Ramos and HA HBL2 cells. Finally, the downregulation of mitochondrial proteins and enzymes was also observed in HA Ramos cells (e.g., COX6C, COX7C, ERAL1, PCCB, DLD), but to a lesser extent compared to HA HBL2 cells.

## Discussion

The major goal of this study was the complex analysis of impact of long-term deep uninterrupted hypoxia on lymphoma cell lines, including gene and protein expression, metabolome, key energy-metabolic pathways, and sensitivity to selected anti-cancer drugs. We are well aware of many weaknesses associated with the in vitro approach of studying hypoxia, but similar approach had been repeatedly exploited by other research groups.

Our data indicate that most (if not all) lymphoma cell lines have difficulties surviving long-term deep uninterrupted hypoxia (1% O_2_, 5% CO_2_) under in vitro culture conditions. However, it is possible (or even plausible) that tumor microenvironment can provide diverse pro-survival stimuli that increase survival of lymphoma cells under in vivo hypoxia, including upregulation of anti-apoptotic molecules (e.g., BCL-XL), or activation of several key prosurvival signaling pathways (e.g., PI3K-AKT) [4]. In contrast to so far published studies, our hypoxia-adapted lymphoma cell lines demonstrated increased sensitivity to most of the tested anti-cancer agents [22, 23]. Therefore, long-term hypoxia appears (at least in vitro) as a clear disadvantage even for hypoxia-adapted lymphoma cells. It is, however, conceivable that in vivo hypoxia may lead to cell cycle arrest and long-term survival of lymphoma cells in deeply hypoxic microenvironments that are out of reach of chemotherapy or cells of the immune system (e.g., a bone marrow osteoblastic niche, a bulky tumor near its necrotic parts, or malignant effusions) [5, 24].

Of note, both cell lines adaptable to long-term uninterrupted hypoxia in this study (i.e., HBL2 and Ramos) represent commercially available cell lines that had been established more than 30 years ago in contrast to the patient-derived cell lines with limited numbers of in vitro passages. It cannot be excluded that during the long periods in culture, HBL2 and Ramos cells underwent additional genetic mutations that made them more robust and adaptable to long term deep hypoxia. Both HA HBL2 and HA Ramos cells had significant downregulation of key energy-metabolic pathways, i.e., oxidative phosphorylation and glycolysis. This functional downregulation was accompanied by markedly decreased levels of most of the evaluated metabolites. Furthermore, both transcriptome and proteome analyses suggested several shared strategies of lymphoma cell adaptation to long-term hypoxia. Long-term hypoxia resulted in marked downregulation of mitochondrial proteins (mainly in HA HBL2 and to a lesser extent in HA Ramos cells), namely mitochondrial ribosomal subunits and components of complex I and IV of ETC, which is consistent with previous studies [21]. Additionally, adaptation to hypoxia was associated with reduction of total mitochondrial DNA (mtDNA) content in both HA cell lines, which was also reported for DLBCL cell lines cultured under hypoxia [25]. Such an obvious depletion of mitochondrial proteins as well as mtDNA suggests possible mitochondrial recycling via mitophagy, a selective form of hypoxia-induced autophagy that removes dysfunctional mitochondria [26, 27]. Hypoxia induced activation of mitophagy was supported also at the transcriptome level as both HA cell lines upregulated mitophagy associated genes, namely *BNIP3*, *BNIP3L*, or *BNIP3P1* [28].

In contrast, upregulation of proteins involved in glucose metabolism was a predominant finding in HA Ramos cells (and to a lesser extent in HA HBL2 cells). These included key glycolysis enzymes (ENOA, ALDOC, PKM, PGAM1, HK1, TPI) as well as glucose transporters (GLUT1/SLC2A1, and GLUT5/SLC2A5). Concurrent upregulation of monocarboxylate transporter 4 (SLC16A3) may also contribute to the putative increase of glucose utilization, as it was reported that SLC16A3 transporter is critically involved in lactate efflux from highly glycolytic cells. Glycolysis represents a critical ATP-producing pathway in hypoxic lymphoma cells [29–31]. Therefore, upregulation of key glycolytic enzymes in response to hypoxia, observed in this and previous studies may be interpreted as an attempt to compensate for functionally decreased glycolytic activity of HA cells [25]. Increased sensitivity to glycolytic inhibitor 2-DG may indicate high if not absolute dependency of HA cells on the residual glycolysis. We have also detected hyperphosphorylation of AKT kinase (a marker of its hyperactivation) in both HA lymphoma cell lines. Therefore, the observed upregulation of genes and proteins involved in glucose metabolism might be mediated by hypoxia (HIF1α)-activated PI3K-AKT pathway [32]. In addition, hyperphosphorylation of AKT was associated with increased sensitivity of HA Ramos cells to copanlisib, a pan-PI3K inhibitor. Several PI3K inhibitors (idelalisib, copanlisib) were approved for the therapy of various lymphoproliferative malignancies, while AKT inhibitors (capivasertib, ipatasertib) were recently approved for clinical use in breast cancer patients [33]. Our data thus suggest a novel mode-of-action of the PI3K/AKT inhibitor class of agents- a preferential elimination of HA cancer cells.

Importantly, using caspase inhibitors we have demonstrated that cytotoxic effects of hypoxia are mediated via apoptosis activation. It is thus not surprising that adaptation to long-term hypoxia was associated with deregulated expression of key proapoptotic (BIM, NOXA, BAD) and antiapoptotic (BCL-XL, MCL1) proteins. In addition, increased binding of BIM to BCL-XL in both HA cell lines was demonstrated by immunoprecipitation experiments. It suggests an increased dependence of HA cells on BCL-XL for survival, as well as priming of HA cells to BCL-XL targeting BH3 mimetics. Indeed, both HA cell lines were significantly more sensitive to A1155463, a nanomolar BCL-XL inhibitor, as compared to their respective normoxic controls. Among other, BCL-XL belongs to critical modulators of sensitivity to venetoclax, a clinically approved BCL2 inhibitor [34]. It was repeatedly demonstrated that BCL-XL is upregulated by hypoxia [35, 36] and this was also observed in our HA Ramos cells. We can only speculate that hypoxia- and microenvironment-induced (over)expression of BCL-XL may be responsible for survival of venetoclax-resistant lymphoma clones, and conversely that co-targeting BCL-XL and BCL2 may prove synergistic in vivo.

Finally, we identified P4HA1, a catalytic component of prolyl 4-hydroxylase, as a novel potential mediator of hypoxia adaptation. P4HA1 was upregulated in both HA cell lines at both levels, transcriptome, and proteome. High expression of P4HA1 negatively correlated with survival in many cancer types [37–41]. Expression of P4HA1 in pancreatic cancer was demonstrated to be dependent on HIF1α and to correlate with hypoxia gene signatures, and glycolysis. P4HA1 in turn enhanced HIF1α stability, indicating a positive feedback loop between HIF1α and P4HA1 in pancreatic cancer [37].

In conclusion, we unveiled several shared molecular strategies leading to adaptation of lymphoma cell lines to long-term deep hypoxia and tested several innovative agents for targeted eradication of hypoxia-adapted malignant lymphocytes.

## Materials and methods

### Establishment of cell lines adapted to long-term hypoxia

Lymphoma cell lines comprised the following NHL subtypes: diffuse large B-cell lymphoma (UPF4D, UPF8D), mantle cell lymphoma (UPF1H, HBL2), and Burkitt lymphoma (Ramos, UPF9T). Ramos was purchased from the German Collection of Microorganisms and Cell Cultures (DSMZ). HBL2 was a kind gift from Prof. Martin Dreyling. UPF4D, UPF8D, UPF9T and UPF1H cell lines were established at the Institute of Pathological Physiology by ex vivo culture of primary lymphoma cells obtained from patients with chemotherapy-refractory aggressive lymphomas as part of other research projects approved by Ethics Committee of the General University Hospital Prague under number 48/18 Grant AZV VES 2019 VFN. Whole exome sequencing (WES) analysis confirmed that the newly derived cell lines shared majority of somatic mutations with the primary lymphoma cells, from which they were derived (Supplementary Table 1, Supplementary Fig. 1). All cell lines were authenticated and tested for Mycoplasma contamination. HA lymphoma cells were derived by long-term (≥4 weeks) culture of lymphoma cells under hypoxic conditions (1% O_2_, 5% CO_2_) in a Coy hypoxic chamber. We used a polymer O_2_ control 3-glove box, equipped with a Coy humidified incubation box (ensuring non-condensing environment), a model AC100 CO_2_ controller, an oxygen controller (regulating direct supply of O_2_, CO_2_, and N_2_), and an ergonomic sliding airlock shelf (enabling cell transfer inside and outside the hypoxic chamber without producing changes in the hypoxic chamber). In the Coy hypoxic chamber, the cells were cultured in the Iscove´s Modified Dulbecco Medium (IMDM) supplemented with 15% Fetal Bovine Serum (FBS) under 37 °C Celsius and 5% CO_2_ in a humidified standard pressure atmosphere. During the derivation of HA cells, the hypoxic conditions were not interrupted, and the fresh medium was supplemented 2–3 times weekly. To achieve that, the cells were placed into a 50 mL centrifuge tube, sealed with a cap and paraffin tape, transferred through the airlock outside the hypoxic chamber, and after centrifugation it was returned (again through the airlock) back to the hypoxic chamber, where the old medium was replaced by the fresh medium that had been placed to the hypoxic chamber for at least 24 h before its use in an empty cultivation flask with a filter (that enabled exchange of the O_2_ and CO_2_ gasses). Both HBL2 and Ramos cells had been cultured continuously for 4 weeks under hypoxia before the cells were designated hypoxia-adapted HBL2 (HA HBL2) or HA Ramos. Only HA HBL2 and HA Ramos cells were subjected to transcriptome, proteome, metabolic, and functional analyses. In all cases, the HA cells cultured under hypoxia (1% O_2_, 5% CO_2_) were compared to the original lymphoma cell lines cultured in parallel under standard “normoxic” oxygen conditions [42].

### Apoptosis and proliferation assays

Cytotoxic agents (cisplatin, cytarabine, bortezomib) were purchased from the Charles University General Hospital pharmacy. Tumor necrosis factor (TNF)-related apoptosis-inducing ligand (TRAIL) was purchased from Exbio Praha a.s., Czech Republic. Venetoclax, a BCL2 inhibitor, S63845, a MCL1 inhibitor, and A1155463, a BCL-XL inhibitor, and IM-156, an inhibitor of oxidative phosphorylation, were purchased from MedChemExpress (via Scintila s.r.o., Czech Republic). Unless specified, all chemicals were from Merck Life Science, Czech Republic.

The cells were exposed to the tested cytotoxic agents (e.g., genotoxic cytostatics, mitochondrial targeting agents, etc.) for 24 h, after which the cells were washed with PBS (VWR Chemicals), resuspended in 1x Annexin binding buffer (Exbio Praha a.s., Czech Republic), and stained with Annexin V FITC (50x diluted) for 15 min. Samples were then 3x diluted with Annexin binding buffer and stained with propidium iodide (Thermo Scientific, c = 8.89 ng/μl). Stained samples were analyzed on NovoCyte Penteon cytometer (Agilent, United States), and the data were analyzed using FlowJo software (version 10.8.1). Conjugated Annexin V was purchased from Exbio Praha a.s. (Czech Republic), a pan-caspase inhibitor Z-VAD-FMK, a broad-spectrum caspase inhibitor Q-VD-OPh, and an inhibitor of necroptosis necrostatin 1 (NEC-1) were purchased from Bio-Techne R&D Systems s.r.o., Czech Republic.

The proliferation assays were implemented as follows: the cells were counted and cultivated under the defined conditions with or without the selected cytotoxic agents. Proliferation rate was measured at the selected timepoints using the WST-8 cell proliferation assay according to the manufacturer´s instructions. WST-8 was from Abcam, Cambridge, United Kingdom, provided by BioTech a.s., the distributor in the Czech Republic.

### Cell cycle analysis

The cell cycles of control and hypoxia-adapted cells were analyzed using the BD Pharmingen ^TM^ APC BrdU Flow Kit (BD Bioscience, Franklin Lakes, NJ, USA) according to the manufacturer’s instructions. Briefly, 2×10^6^ cells were cultured for 45 min in 2 mL of IMDM supplemented with 15% FBS, 1% penicillin-streptomycin (all from Biosera, Cholet, France) and 10 μM 5-bromo-2’-deoxyuridine (BrdU) in either normoxia or hypoxia (1% O_2_) and subsequently fixed and cryopreserved [42]. Upon thawing, the samples were refixed and treated with DNase (300 μg/mL in PBS) followed by a wash cycle (300 g, 4 °C, 5 min). The pellet was resuspended in 50 μL of PBS and stained with an APC-conjugated anti-BrdU antibody (Sony Biotechnology, San Jose, CA, USA) at the final concentration of 52 μg/mL to visualize newly synthesized DNA. Following another wash cycle, the cells were stained with 7-AAD at the final concentration of 10 ug/mL to visualize total DNA. Prepared samples were analyzed using FACS Canto II cytometer (BD Bioscience, Franklin Lakes, NJ, USA). Data analysis was performed using FlowJo v10 and GraphPad Prism 9 software.

### Measuring of mitochondrial DNA

For quantitative polymerase chain reaction (qPCR), cells were harvested, and total DNA was isolated from HA lymphoma cells cultured under hypoxia, and from the corresponding controls cultured under normoxia using DNeasy Blood & Tissue Kit (Qiagen). Primers for mitochondrial sequences MajorArc and MinorArc were synthesized by Sigma Aldrich [43]. Quantitative PCR (qPCR) was performed using HOT FIREPol® EvaGreen® qPCR Supermix, 5x (Solis Biodyne), the reaction was pipetted according to the manufacturer’s protocol, qPCR was run on a BioRad cycler, the measured data of the mitochondrial sequences MajorArc and MinorArc were analyzed in MS Excel, 2^Ct value was calculated and subsequently the data were initially normalized to levels of b-2 microglobulin representing nuclear DNA (nDNA) and these values then to “normoxic control = 1” for each cell line [43].

### Next generation transcriptome sequencing

Total RNA was isolated using RNeasy® Mini Kit (Qiagen, Germany) according to the manufacturer’s protocol. Quality of RNA was confirmed by Agilent RNA 6000 Nano Kit (Agilent Technologies, USA).

RNA-Seq libraries were prepared using Agilent SureSelect mRNA Strand Specific kit (Agilent Technologies, CA, USA) and paired-end (2x75bp) sequenced on NextSeq 500 (Illumina, USA). QC of the FASTQ files and trimming was performed using TrimGalore. Read pairs were mapped to the hg19 reference genome with TopHat2 and counts obtained using HTSeq [44, 45]. Downstream differential expression analysis was performed by fitting a negative binomial generalized log-linear model to the read counts for each gene using EdgeR package [46]. The matrix of counts was filtered by expression and the library sizes were normalized using trimmed mean of M-values approach. Remaining low expressed transcripts were removed using a threshold of more than 0.5 counts per million transcripts in at least two samples. To test for differentially expressed genes, we estimated trended and tagwise dispersions, fitted negative binominal generalized linear model and applied quasi-likelihood F-test. Genes were considered differentially expressed at fold change more than 2 and with *p* value less than 0.05. Gene set enrichment analysis was conducted on lists of differentially expressed genes ranked by log2 fold change using ReactomePA R package [47]. *P* values were adjusted using the Benjamini-Hochberg procedure and cut off at 0.05.

### Proteome analysis

#### Sample preparation

Dry cell pellets (30 mg) were lysed, reduced, alkylated, and digested with trypsin. The peptides were labeled with 12 tags from the TMTpro™ 16plex Label Reagent Set (Thermo Scientific™, Massachusetts, USA) according to the manufacturer’s instructions. Labeled peptides were combined, desalted and dried before the LC-MS/MS analysis. For more detailed description, see Supplementary methods.

#### 2D-LC-MS/MS analysis

The TMT-labeled peptide sample was first fractionated on a reverse phase at high pH. Sixty-four fractions were collected and combined into 8 pooled fractions. Each fraction was then separated and analyzed by nanoHPLC Dionex Ultimate 3000RS connected to Thermo Orbitrap Fusion. The MS2 spectra were measured in an ion trap with CID fragmentation, the MS3 spectra (quantitation) were collected in the Orbitrap with HCD fragmentation. The obtained raw data were searched with Proteome Discoverer 2.4 with Sequest search engine. FDR was set up at 0.01 for both peptide and protein. Only the proteins identified with at least two unique peptides were considered. For more detailed descriptions, see Supplementary methods.

### Metabolomics analysis

The cell lysates were prepared in a manner suitable for a LC-MS measurement. The cells were counted and harvested in triplicates. The pellets were washed in 137 mM NaCl + 2.7 mM KCl buffer three times and kept on ice and the metabolites were extracted with 1 ml of extraction solution (4:4:2, methanol:acetonitril:water with 0.1 M formic acid) with an addition of an internal standard (2 μg/ml ribitol), followed by vortexing for 15 s and centrifugation at 15,000 × *g*, 4 °C, 10 min. The supernatant was evaporated and resuspended in final 50 μl of 50% acetonitrile containing internal standards (3.3 μM 1,4-piperazinediethanesulfonic acid, 1.5 μM ^15^N_5_-AMP). The samples were analyzed on a Dionex Ultimate 3000RS liquid chromatography system coupled to a TSQ Quantiva mass spectrometer (Thermo Scientific). Data were processed using the Skyline software. For more information, see Supplementary Methods.

### Oxygen consumption rate (OCR) and extracellular acidification rate (ECAR) assays

Oxygen consumption rate (OCR) and extracellular acidification rate (ECAR) evaluations were performed using the Seahorse XFe 96 analyzer (Agilent Technologies, Santa Clara, California, USA). For more information, see Supplementary Methods.

### Western blotting and immunoprecipitation

Western blotting and immunoprecipitation experiments (with anti-BCL-XL antibody) were implemented as previously described [34]. The list of antibodies used in this project is displayed in Supplementary Table 2.

### Whole exome sequencing analysis of new lymphoma cell lines

Tumor and derived cell line genomic DNA were extracted using DNeasy Blood & Tissue Kit (Qiagen, Germany) and sequenced using Illumina NextSeq 500. The reads were aligned to hg38 using BWA and somatic variants were called using Genome Analysis Toolkit. For more information, see Supplementary Methods.

### Statistical analyses

Student´s *t* test was used to assess statistical significance of the experiments analyzing apoptosis, cell cycle, or qPCR results. Statistical analysis of the OMICs data (i.e., transcriptome, proteome, and metabolome) are specified in detail in the respective sections of the methods.

### Ethics approval and consent to participate’ statement

All methods were performed in accordance with the relevant guidelines and regulations. UPF4D, UPF8D, UPF9T and UPF1H cell lines were established at the Institute of Pathological Physiology by ex vivo culture of primary lymphoma cells obtained from patients with chemotherapy-refractory aggressive lymphomas after obtaining informed consent as part of other research projects approved by Ethics Committee of the General University Hospital Prague under number 48/18 Grant AZV VES 2019 VFN.

## Supplementary information

Suplemental Data File

Supplemental Western Blots

## Data Availability

All data generated in this manuscript are fully available either as part of the Supplemental Data File, or via hyperlinks to datasets uploaded to publicly accessible repositories.
