# Supplementary material for: Long-term adaptation of lymphoma cell lines to hypoxia is mediated by diverse molecular mechanisms that are targetable with specific inhibitors
Source: Cell Death Discov. 2025 Feb 18;11:65. doi: 10.1038/s41420-025-02341-y (PMC11836139; doi:10.1038/s41420-025-02341-y)
Supplement: Supplementary file 1 — Suplemental Data File [file 41420_2025_2341_MOESM1_ESM.docx]

**SUPPLEMENTAL DATA FILE**

***Long-term adaptation of lymphoma cell lines to hypoxia is mediated by diverse molecular mechanisms that are targetable with specific inhibitors***

**Running Title:** Adaptation of lymphomas to long-term hypoxia

**Authors:** Lenka Daumova^1^, Dmitry Manakov^1^, Jiri Petrak^2^, Dana Sovilj^3^, Matěj Behounek^2^, Ladislav Andera^3,4^, Ondrej Vit^2^, Olga Souckova^5^, Ondrej Havranek^2,6^, Nicol Renesova^1^, Alex Dolnikova^1^, Liliana Tuskova^1,6^, Lucie Winkovska^7^, Nardjas Bettazova^1,8^, Kristina Kupcova^2,6^, Marie Hubalek-Kalbacova^1^, Miriama Sikorova^1^, Marek Trneny^6^, and Pavel Klener^1,6*^

**Corresponding author*

**Affiliations:** ^1^Institute of Pathological Physiology, First Faculty of Medicine, Charles University, Prague, Czech Republic; ^2^BIOCEV Biotechnology and Biomedicine Centre, First Faculty of Medicine, Charles University, Vestec, Czech Republic; ^3^Institute of Biotechnology BIOCEV, Czech Academy of Sciences, Prague, Czech Republic; ^4^Institute of Molecular Genetics, Czech Academy of Sciences, Prague, Czech Republic; ^5^OMICS Mass Spectrometry Core Facility, Biology Departments, BIOCEV, Faculty of Science, Charles University; ^6^First Department of Medicine- Hematology, University General Hospital Prague and First Faculty of Medicine, Charles University, Prague, Czech Republic; ^7^CLIP, Department of Pediatric Hematology/Oncology, Second Faculty of Medicine and University Hospital in Motol, Prague, Czech Republic; ^8^Department of Medical Genetics, Third Faculty of Medicine, Charles University, Prague, Czech Republic

**Corresponding author:**

Prof. Pavel Klener, M.D., Ph.D., Institute of Pathological Physiology, U Nemocnice 5, Prague 2, 12853, Czech Republic; and First Dept. of Internal Medicine- Hematology, University General Hospital in Prague and First Faculty of Medicine, Charles University, U Nemocnice 2, 12808, Prague 2, Czech Republic; e-mail: pavel.klener2@lf1.cuni.cz

Tel: +420-224 965 993; Fax: +420-224 965 916

ORCID: 0000-0001-7786-9378

**Supplemental Methods**

***Proteomic analysis***

***Sample preparation and labelling***

Dry cell pellets (30mg) were lysed at room temperature for 30 minutes in 500µl of lysis buffer containing 5% sodium deoxycholate and 100 mM TEAB (100mM, pH 8.5) and sedimented at 20,000× g for 30 minutes. Supernatants were collected, and the protein concentration was determined using a BCA assay (Sigma-Aldrich, Darmstadt, DE). Samples representing 60µg of protein were diluted with 100mM TEAB to final volume of 100µl, reduced by 5µl of 200 mM TCEP at 55°C for one hour and alkylated by 5µl of 375mM iodoacetamide at room temperature in dark for 30 minutes. The alkylated proteins were precipitated overnight at -20°C by 600µl of pre-chilled acetone. The precipitate was recovered at 8,000× g for 10 minutes at 4°C.

Dried protein pellets were resuspended in 100µg of 100mM TEAB and digested with trypsin (Promega, 1.5 µg per 60µg protein) overnight at 37°C. The first twelve TMT labels from the TMTpro™ 16plex Label Reagent Set (Thermo Scientific™, Massachusetts, USA) were used according to the manufacturer’s instructions, downscaled to 60µg of proteins per sample. The twelve TMT labels were assigned to the sample triplicates as follows: “HBL2 normoxia” (labels: 126/ 127N, 127C), “HBL2 1% O2 adaptation” (labels: 128N/ 128C/ 129N), “Ramos normoxia” (labels: 129C/ 130N/ 130 C), and “Ramos 1% O2 adaptation” (labels: 131N/ 131C/ 132N). Half the volume of each solubilized TMT label was used for each protein sample, labelling was done on a platform shaker at 300 RPM for one hour at room temperature. Finally, the reaction process was stopped by introducing 8µl of 5% hydroxylamine for 15 minutes. The labelled samples were combined into a single tube and desalted through manually operated Opti-trap cartridge C18 column (Optimize Technologies, Orgeon City, OR, USA). Column equilibration and washing was done in 0.1% TFA, and a solution of 80% acetonitrile in 0.1% TFA was used for the peptide elution. Desalted sample was dried using a SpeedVac Vacuum Concentrator (Thermo Scientific™, Massachusetts, USA).

***2D-LC-MS/MS analysis***

The TMT-labelled peptide sample was first fractionated on a reverse phase at high pH using YMC column (YMC, Japan) (300 mm, 0.3 mm, 1.9 µm). Linear 60 min gradient was applied 2 µl/min, (A – 20 mM NH4FA, 2% ACN; B – 20 mM NH4FA, 80% ACN) from 1% B to 60% B. Sixty-four fractions were collected and combined into 8 pooled fractions by the farthest neighbor method.

Each fraction was resuspended in 1% TFA in 2% ACN and 1.5 µg of peptide was injected onto nanoHPLC Dionex Ultimate 3000RS connected to Thermo Orbitrap Fusion. Thermo PepMap Trap column (160454) was used for peptide pre-concentration and the separation was done on 50 cm EASY Spray Column (Thermo Scientific, ES903) using 180 min gradient from 2% A to 35% B (A:0.1% FA, B:99.9% CAN, 0.1% FA). Cycle time was set to 4 s. The MS2 spectra for identification were measured in an ion trap with CID fragmentation, 60 ms maximum injection time, the MS3 spectra (quantitation) were measured in the Orbitrap with HCD fragmentation using the SPS function, 118 ms maximum injection time, 10 precursors for synchronous precursors selection feature.

The obtained raw data were searched with Proteome Discoverer 2.4 with Sequest search engine. The method was based on a predefined workflow for SPS MS3 isobaric quantification with the batch specific correction parameters for TMT 16plex label were used. Searches were performed using a database of Human – Uniprot reviewed (Release 2021_04) along with common contaminants database. Dynamic modifications were set as follows: Oxidation/ +15.995 Da (M), protein N-terminal acetyl/ +42.011 Da, Met-loss/-131.040 Da (M), Met-loss+Acetyl/-89.030 Da (M). Static Modifications: Carbamidomethyl/+57.021 Da (C), TMT6plex/+229.163 Da (K), Peptide N-Terminus: TMT6plex /+229.163 Da. FDR was set up at 0.01 for both peptide and protein using Perolator. For reporter ions detection 20 pmm tolerance was set with most confident centroid peak integration. Only the proteins identified with at least two unique peptides were considered.

***Metabolomics analysis***

The samples were analyzed on a Dionex Ultimate 3000RS liquid chromatography system coupled to a TSQ Quantiva mass spectrometer (Thermo Scientific) using electrospray ionization in switching polarity mode with the following ion source parameters: ion transfer tube temperature 325 °C, vaporizer temperature 275 °C, spray voltage 3500/2800 V (positive/negative mode), sheath gas 35, and aux gas 7. A SeQuant® ZIC®-pHILIC column (150 mm × 2.1 mm, 5 µm) coupled with a SeQuant® ZIC®-pHILIC Guard (20 mm × 2.1 mm) (Merck, Darmstadt, Germany) was used for analyte separation. The buffer composition was A: 10 mM ammonium bicarbonate pH 9.3 and B: 97% acetonitrile, at a flow rate of 200 µL/min. The elution gradient (A/B) used was 0 – 2.5 min: 5% A; 2.5 – 21 min: 5% A to 70% A; followed by washing phase 21 – 23 min: 70 % A and equilibration phase 23.1 – 32 min: 5% A. Selective reaction monitoring (SRM) was used for the detection and quantification of selected compounds. Data were processed using the Skyline software.

***Oxygen Consumption Rate (OCR) and Extracellular Acidification Rate (ECAR) assays***

HA lymphoma cell lines cultured under hypoxia (1% O_2_) and the original lymphoma cell lines cultured under normoxia were seeded in Corning Cell-Tak-coated XFe 96 cell culture microplates (4x10^4^ cells/well, Agilent Technologies) on the day of the experiment in the Seahorse base medium (Agilent Technologies) and incubated at 37 °C for 1 h. The Seahorse base medium (180 μl/well) was supplemented with 500 mM pyruvate, 2 mM L-glutamine, and 10 mM glucose for OCR measurements, or with 2 mM L-glutamine for ECAR measurements. Prior to the measurement cells were incubated at 37 °C without CO_2_ for 30 minutes. The Mito Stress Test was run employing series of injections, starting with oligomycin (1 μM, inhibits ATP synthase, port A), followed by carbonyl cyanide 4-(trifluoromethoxy) phenylhydrazone (FCCP, 2 μM, mitochondrial uncoupler, port B), and a combination of antimycin and rotenone (0.5 μM, inhibitors of CIII and CI, port C). The glycolytic stress test was performed by injection of glucose (10 mM, substrate of glycolysis, port A), followed by oligomycin (1 μM, port B), followed by 2-deoxy-D-glucose (2DG, 50 mM, glycolysis inhibitor, port C). OCR and ECAR readings were taken after each injection. After the assays, cells were stained with Hoechst 33342 and counted using the Cytation5 (Agilent Technologies) instrument. OCR and ECAR values were normalized based on the cell number.

***Whole exome sequencing (WES) of newly derived lymphoma cell lines***

All newly derived lymphoma cell lines (i.e., UPF4D, UPF8D, UPF1H, and UPF9T) were established by *ex vivo* culture of primary lymphoma cells obtained from patients with relapsed / refractory B-cell non-Hodgkin lymphomas as part of other research projects approved by Ethics Committee of the General University Hospital Prague under number 48/18 Grant AZV VES 2019 VFN (Supplemental Table 1). The mutational profiling implemented by whole exome sequencing (WES) confirmed that the newly derived lymphoma cell lines shared majority of somatic mutations with the primary lymphoma cells from which they were derived (Supplemental Figure 1).

Genomic DNA was extracted from the fresh frozen cells using DNeasy Blood & Tissue Kit (Qiagen, Germany) according to the manufacturer’s protocol. To filter out gene polymorphisms present in tumor DNA, non-tumorous (germline) DNA was obtained from each patient either from a buccal smear or from a bone marrow sample (with no detectable lymphoma infiltration by flow cytometry) obtained by trephine. All tumor tissue samples included in the WES analysis had tumorous infiltration ≥30% according to flow cytometry.

Samples were sequenced on the NextSeq 500 instrument (Illumina, San Diego, CA) according to the manufacturer’s protocols with sequencing libraries prepared using SureSelectXT Human All Exon V8 + UTR kit (Agilent Technologies, Santa Clara, CA). The resulting reads were then aligned against the human reference genome hg38. The alignments were performed using BWA (version 0.7.17)^1^. Somatic variants were called and annotated using Genome Analysis Toolkit (GATK) release 4.6.0.0^2^. According to the best practices, we marked the duplicate reads, applied the base quality recalibration, and performed somatic variant calling using Mutect2^3^. Resulting variants were filtered and annotated using FilterMutectCalls and Funcotator tools respectively. The variants were additionally filtered to exclude those with allele frequency in tumor sample less than 5 % and manually curated using Integrative Genome Viewer to exclude those with less than 3 observed reads^4^.

***References***

1. Li, H. & Durbin, R. Fast and accurate short read alignment with Burrows-Wheeler transform. *Bioinforma. Oxf. Engl.* **25**, 1754–1760 (2009).

2. Auwera, G. A. V. de & O’Connor, B. D. *Genomics in the Cloud: Using Docker, GATK, and WDL in Terra*. (O’Reilly, Beijing Boston Farnham Sebastopol Tokyo, 2020).

3. Van der Auwera, G. A. *et al.* From FastQ Data to High-Confidence Variant Calls: The Genome Analysis Toolkit Best Practices Pipeline. *Curr. Protoc. Bioinforma.* **43**, 11.10.1-11.10.33 (2013).

4. Robinson, J. T. *et al.* Integrative Genomics Viewer. *Nat. Biotechnol.* **29**, 24–26 (2011).

**Supplemental Tables and Figures**

**Supplemental Table 1.** *Basic characterization of the newly derived lymphoma cell lines.*

| **Cell line** | **Patient** | **Disease** | **Source of primary cells** | **Disease status** | **Previous lines of therapy** |
| --- | --- | --- | --- | --- | --- |
| **UPF1H** | P001 | MCL | Leukemized blood | Treatment Refractory | 1 |
| **UPF4D** | P005 | GCB-DLBCL | Malignant ascites | Treatment Refractory | 2 |
| **UPF8D** | P013 | GCB-DLBCL | Malignant ascites | Treatment Refractory | 2 |
| **UPF9T** | P016 | Burkitt | Infiltrated bone marrow | Treatment Refractory | 1 |

**Table 1 legend:** MCL = mantle cell lymphoma, GCB-DLBCL= germinal B-cell diffuse large B-cell lymphoma; at the time of cell line derivation, all patients had been treated with the combination of chemotherapy and anti-CD20 antibody, and were refractory to the last therapy.

**
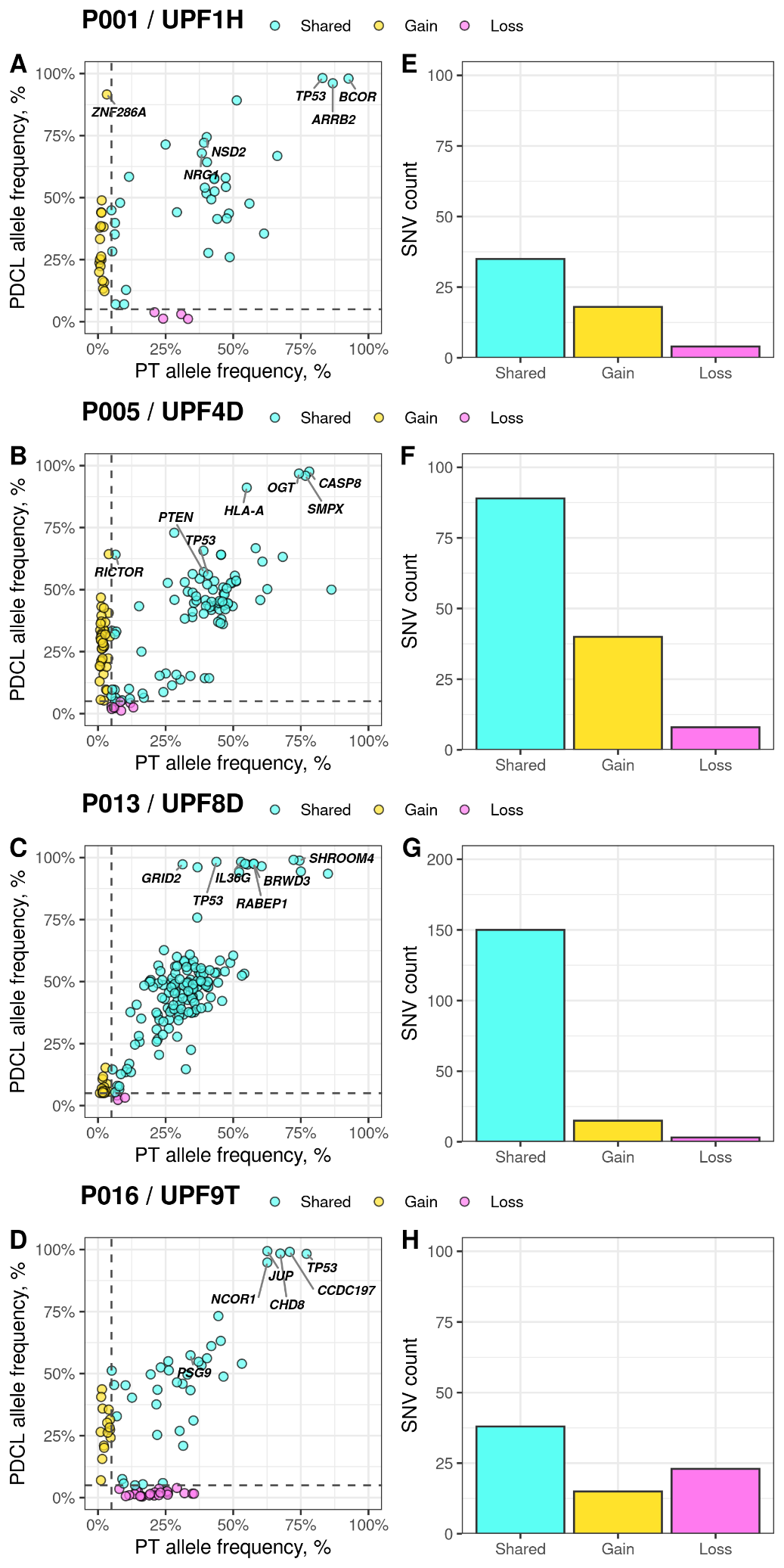
**

**Supplemental Figure 1.** *Mutational profiling of newly derived lymphoma cell lines by whole-exome sequencing: head-to-head comparison with the respective primary lymphoma cells*

**Supplemental Figure 1 legend:** Single nucleotide variants (SNVs) detected by WES analysis of the newly derived lymphoma cell lines (UPF1H, UPF4D, UPF8D, and UPF9T) and the original primary lymphoma cells obtained from patients with relapsed or refractory lymphomas (P001, P005, P013, and P016). **A-D:** scatterplots of the filtered SNVs; *X* and *Y* axis shows variant allele frequency of the filtered SNVs in the primary lymphoma cells and lymphoma cell lines, respectively;, **E-H:** bar plots show numbers of the filtered SNVs detected both in the primary lymphoma cells and lymphoma cell lines (“shared”, blue), SNVs detected in the lymphoma cell lines, but not in the primary lymphoma cells (“gained”, yellow), and SNVs detected in the primary lymphoma cells, but not in the lymphoma cell lines (“lost”, purple). Detailed characterization of the filtered SNVs can be downloaded from https://doi.org/10.5281/zenodo.10993246.

**
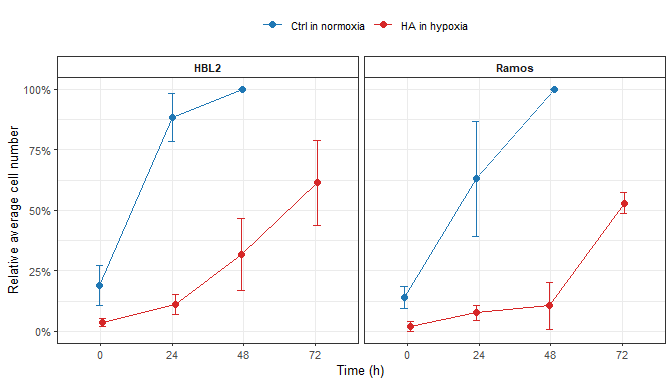
**

**Supplemental Figure 2.** *Significantly slower proliferation rate of HA lymphoma cells compared to the corresponding controls growing under normoxic conditions.* Proliferation rate of HA lymphoma cells cultured under long-term (i.e., > 4 weeks) hypoxia (red, 1% O2) compared to the original lymphoma cell lines cultured under normoxia (blue).

**Supplemental Table 2.** *List of antibodies used for western blotting and immunoprecipitation.*

| **Acrylamide %** | **Target** | **Antibody supplier** | **Cat. #** | **kDa** | **IgG source** | **Dilution** |
| --- | --- | --- | --- | --- | --- | --- |
| 12 | α-tubulin | Abcam | ab7291 | 50 | mouse | 1:5000 |
| 15 | BCL-2 | CST | 610539 | 28 | mouse | 1:5000 |
| 12 | MCL-1 | CST | 39224 | 40 | rabbit | 1:2000 |
| 15 | BCL-XL | CST | 2764S | 30 | rabbit | 1:5000 |
| 15 | BCL2L11/BIM | CST | 2933 | 12, 15, 25 | rabbit | 1:2000 |
| 15 | NOXA | Abcam | ab13654 | 10 | mouse | 1:2000 |
| 12 | BAD | CST | 9292 | 23 | rabbit | 1:2000 |
| 15 | pBAD(S136) | CST | 9295 | 23 | rabbit | 1:1000 |
| 12 | P4HA1 | Proteintech | 12658-1-AP | 60 | rabbit | 1:2000 |
| 12 | AKT | CST | #9272 | 60 | rabbit | 1:2000 |
| 12 | p-AKT | CST | #4060 | 60 | rabbit | 1:500 |

**Abbreviations:** BCL-2 = B-cell lymphoma 2, CST = Cell Signaling Technology

**Supplemental Table 3.** *Fold change and adjusted P values for metabolites detected in HA HBL2 compared to normoxic HBL2. Ribitol was used as an internal standard.*

| **Metabolite** | **Mean (fold change)** | **Adjusted P value** |
| --- | --- | --- |
| glutathione oxidized | 0.29 | 0.012 |
| isocitric acid | 0.11 | 0.014 |
| ADP | 0.45 | 0.017 |
| aconitic acid | 0.14 | 0.017 |
| citric acid | 0.20 | 0.017 |
| succinic acid | 0.28 | 0.017 |
| pyruvic acid | 2.36 | 0.022 |
| prolin | 0.61 | 0.022 |
| cytosine | 0.13 | 0.025 |
| valine | 0.82 | 0.025 |
| ATP | 0.36 | 0.026 |
| serine | 2.03 | 0.026 |
| citrulline | 1.84 | 0.041 |
| fumaric acid | 0.57 | 0.046 |
| aspartate | 1.54 | 0.046 |
| glutathione reduced | 0.56 | 0.047 |
| malic acid | 0.59 | 0.052 |
| glutamine | 1.38 | 0.056 |
| AMP | 0.34 | 0.060 |
| acetyl coenzyme A | 0.63 | 0.078 |
| histidine | 0.68 | 0.081 |
| lactic acid | 0.69 | 0.127 |
| gamma-aminobutyric acid | 0.82 | 0.159 |
| ornithine | 1.44 | 0.159 |
| arginine | 0.64 | 0.195 |
| tyrosine | 0.93 | 0.197 |
| fructose-1,6-bisphosphate | 0.85 | 0.211 |
| nicotinamide adenine dinucleotide | 0.89 | 0.221 |
| 2-hydroxyglutaric acid | 1.14 | 0.291 |
| alanine | 0.87 | 0.378 |
| leucine | 0.98 | 0.378 |
| isoleucine | 0.92 | 0.407 |
| tryptophan | 0.93 | 0.453 |
| glutamic acid | 0.93 | 0.484 |
| asparagine | 0.95 | 0.578 |
| lysine | 0.88 | 0.594 |
| 2-oxoglutaric acid | 0.95 | 0.728 |
| nicotinamide adenine dinucleotide phosphate | 1.03 | 0.800 |
| glycine | 1.03 | 0.823 |
| threonine | 1.02 | 0.902 |
| phenylalanine | 1.00 | 0.926 |

**Supplemental Table 4.** *Fold change and adjusted P values for metabolites detected in HA HBL2 compared to normoxic HBL2. Ribitol was used as an internal standard.*

| **Metabolite** | **Mean (fold change)** | **Adjusted P value** |
| --- | --- | --- |
| ATP | 0.06 | 0.012 |
| fumaric acid | 0.24 | 0.012 |
| ADP | 0.09 | 0.014 |
| asparagine | 0.31 | 0.017 |
| citric acid | 0.03 | 0.017 |
| lactic acid | 0.09 | 0.017 |
| malic acid | 0.21 | 0.017 |
| nicotinamide adenine dinucleotide | 0.34 | 0.017 |
| proline | 0.17 | 0.017 |
| threonine | 0.34 | 0.017 |
| tryptophane | 0.30 | 0.017 |
| valine | 0.31 | 0.017 |
| aconitic acid | 0.05 | 0.017 |
| alanine | 0.23 | 0.017 |
| serine | 0.50 | 0.019 |
| tyrosine | 0.30 | 0.022 |
| glutamine | 1.93 | 0.024 |
| leucine | 0.29 | 0.024 |
| glutathione reduced | 0.28 | 0.025 |
| 2-oxoglutaric acid | 0.16 | 0.025 |
| cytosine | 0.24 | 0.026 |
| gamma-aminobutyric acid | 0.41 | 0.026 |
| glutamic acid | 0.46 | 0.026 |
| glycine | 0.21 | 0.026 |
| isocitric acid | 0.03 | 0.026 |
| AMP | 0.24 | 0.027 |
| citrulline | 0.20 | 0.027 |
| 2-hydroxyglutaric acid | 0.34 | 0.028 |
| lysine | 0.63 | 0.037 |
| pyruvic acid | 0.15 | 0.039 |
| isoleucine | 0.27 | 0.040 |
| glutathione oxidized | 0.48 | 0.042 |
| histidine | 0.52 | 0.050 |
| aspartate | 0.49 | 0.055 |
| arginine | 0.57 | 0.074 |
| succinic acid | 0.19 | 0.122 |
| nicotinamide adenine dinucleotide phosphate | 0.18 | 0.127 |
| ornithine | 1.23 | 0.159 |
| phenylalanine | 0.45 | 0.159 |
| cystine | 1.24 | 0.686 |

**Supplemental Table 5.** *Transcripts upregulated in HA HBL2 cells compared to the normoxic controls (highlighted in grey are proteins upregulated in both HA cell lines) with fold change > 2 and P value < 0.00001*

| **Ensembl** | **Gene Name** | **Description** | **Fold change** | **P value** | **FDR** |
| --- | --- | --- | --- | --- | --- |
| ENSG00000266976 | LOC102724908 | novel transcript | 11.99 | 5.39E-10 | 3.60E-06 |
| ENSG00000225756 | DBH-AS1 | DBH antisense RNA 1 | 7.46 | 4.13E-07 | 2.29E-04 |
| ENSG00000213700 | RPL17P50 | ribosomal protein L17 pseudogene 50 | 7.24 | 4.51E-06 | 1.12E-03 |
| ENSG00000255760 | LINC02422 | long intergenic non-protein coding RNA 2422 | 6.53 | 5.17E-06 | 1.16E-03 |
| ENSG00000246982 | BRPF3-AS1 | BRPF3 antisense RNA 1 | 6.30 | 2.75E-07 | 1.77E-04 |
| ENSG00000114270 | COL7A1 | collagen type VII alpha 1 chain | 5.18 | 3.50E-07 | 2.02E-04 |
| ENSG00000206885 | SNORA75 | small nucleolar RNA, H/ACA box 75 | 5.17 | 3.64E-08 | 5.04E-05 |
| ENSG00000146592 | CREB5 | cAMP responsive element binding protein 5 | 5.07 | 4.01E-09 | 9.26E-06 |
| ENSG00000090104 | RGS1 | regulator of G protein signaling 1 | 5.05 | 1.24E-09 | 3.60E-06 |
| ENSG00000225205 | PDK1-AS1 | PDK1 And ITGA6 Antisense RNA 1 | 4.85 | 4.90E-06 | 1.15E-03 |
| ENSG00000207280 | SNORD20 | small nucleolar RNA, C/D box 20 | 4.60 | 5.09E-06 | 1.16E-03 |
| ENSG00000251432 | LINC02615 | long intergenic non-protein coding RNA 2615 | 4.46 | 8.26E-07 | 3.62E-04 |
| ENSG00000152582 | SPEF2 | sperm flagellar 2 | 4.00 | 5.84E-06 | 1.28E-03 |
| ENSG00000122884 | P4HA1 | prolyl 4-hydroxylase subunit alpha 1 | 3.92 | 3.70E-10 | 3.60E-06 |
| ENSG00000124785 | NRN1 | neuritin 1 | 3.90 | 4.67E-08 | 5.89E-05 |
| ENSG00000229855 |  | novel transcript | 3.82 | 6.47E-08 | 6.90E-05 |
| ENSG00000143479 | DYRK3 | dual specificity tyrosine phosphorylation regulated kinase 3 | 3.74 | 2.82E-07 | 1.77E-04 |
| ENSG00000213190 | MLLT11 | MLLT11 transcription factor 7 cofactor | 3.50 | 2.97E-06 | 8.07E-04 |
| ENSG00000117707 | PROX1 | prospero homeobox 1 | 3.24 | 1.34E-06 | 5.31E-04 |
| ENSG00000232053 |  | novel transcript | 3.21 | 2.64E-06 | 7.47E-04 |
| ENSG00000114200 | BCHE | butyrylcholinesterase | 3.21 | 3.50E-08 | 5.04E-05 |
| ENSG00000099204 | ABLIM1 | actin binding LIM protein 1 | 3.17 | 1.05E-09 | 3.60E-06 |
| ENSG00000231890 | DARS1-AS1 | DARS1 antisense RNA 1 | 3.16 | 7.81E-06 | 1.66E-03 |
| ENSG00000102755 | FLT1 | fms related receptor tyrosine kinase 1 | 3.07 | 3.65E-06 | 9.73E-04 |
| ENSG00000168843 | FSTL5 | follistatin like 5 | 3.02 | 6.66E-07 | 3.18E-04 |
| ENSG00000170873 | MTSS1 | MTSS I-BAR domain containing 1 | 2.95 | 9.66E-08 | 9.57E-05 |
| ENSG00000206530 | CFAP44 | cilia and flagella associated protein 44 | 2.82 | 1.40E-06 | 5.40E-04 |
| ENSG00000164543 | STK17A | serine/threonine kinase 17a | 2.79 | 1.99E-07 | 1.45E-04 |
| ENSG00000241679 |  | novel transcript | 2.72 | 8.17E-06 | 1.66E-03 |
| ENSG00000154721 | JAM2 | junctional adhesion molecule 2 | 2.65 | 8.38E-06 | 1.66E-03 |
| ENSG00000042493 | CAPG | capping actin protein, gelsolin like | 2.64 | 9.94E-06 | 1.86E-03 |
| ENSG00000146278 | PNRC1 | proline rich nuclear receptor coactivator 1 | 2.55 | 1.22E-07 | 1.13E-04 |
| ENSG00000198157 | HMGN5 | high mobility group nucleosome binding domain 5 | 2.41 | 2.02E-06 | 6.35E-04 |
| ENSG00000118432 | CNR1 | cannabinoid receptor 1 | 2.36 | 2.17E-07 | 1.50E-04 |
| ENSG00000205413 | SAMD9 | sterile alpha motif domain containing 9 | 2.33 | 4.97E-07 | 2.55E-04 |
| ENSG00000260822 |  | novel transcript | 2.30 | 4.67E-06 | 1.13E-03 |
| ENSG00000030419 | IKZF2 | IKAROS family zinc finger 2 | 2.28 | 1.65E-06 | 5.70E-04 |
| ENSG00000138764 | CCNG2 | cyclin G2 | 2.28 | 1.26E-06 | 5.14E-04 |
| ENSG00000115548 | KDM3A | lysine demethylase 3A | 2.24 | 8.86E-07 | 3.72E-04 |
| ENSG00000152256 | PDK1 | pyruvate dehydrogenase kinase 1 | 2.24 | 8.34E-07 | 3.62E-04 |
| ENSG00000188994 | ZNF292 | zinc finger protein 292 | 2.23 | 4.57E-07 | 2.44E-04 |
| ENSG00000133739 | LRRCC1 | leucine rich repeat and coiled-coil centrosomal protein 1 | 2.20 | 2.28E-06 | 6.73E-04 |
| ENSG00000175105 | ZNF654 | zinc finger protein 654 | 2.18 | 2.24E-06 | 6.73E-04 |
| ENSG00000104765 | BNIP3L | BCL2 interacting protein 3 like | 2.18 | 7.21E-06 | 1.56E-03 |
| ENSG00000204186 | ZDBF2 | zinc finger DBF-type containing 2 | 2.18 | 4.36E-06 | 1.10E-03 |
| ENSG00000112715 | VEGFA | vascular endothelial growth factor A | 2.11 | 9.10E-06 | 1.78E-03 |
| ENSG00000166123 | GPT2 | glutamic--pyruvic transaminase 2 | 2.10 | 1.77E-06 | 5.70E-04 |
| ENSG00000151135 | TMEM263 | transmembrane protein 263 | 2.06 | 7.98E-06 | 1.66E-03 |
| ENSG00000047644 | WWC3 | WWC family member 3 | 2.01 | 9.76E-06 | 1.85E-03 |

**Supplemental Table 6.** *Transcripts downregulated in HA HBL2 cells compared to the normoxic controls (highlighted in grey are proteins downregulated in both HA cell lines) with fold change < 0.5 and P value < 0.00001.*

| **Ensembl** | **Gene Name** | **Description** | **Fold change** | **P value** | **FDR** |
| --- | --- | --- | --- | --- | --- |
| ENSG00000103021 | CFAP263 | cilia and flagella associated protein 263 | 0.12 | 3.19E-07 | 1.92E-04 |
| ENSG00000166689 | PLEKHA7 | pleckstrin homology domain containing A7 | 0.15 | 5.65E-08 | 6.53E-05 |
| ENSG00000170421 | KRT8 | keratin 8 | 0.17 | 6.26E-07 | 3.10E-04 |
| ENSG00000148180 | GSN | gelsolin | 0.18 | 1.30E-09 | 3.60E-06 |
| ENSG00000149781 | FERMT3 | FERM domain containing kindlin 3 | 0.19 | 9.03E-09 | 1.79E-05 |
| ENSG00000119698 | PPP4R4 | protein phosphatase 4 regulatory subunit 4 | 0.23 | 4.35E-06 | 1.10E-03 |
| ENSG00000133106 | EPSTI1 | epithelial stromal interaction 1 | 0.24 | 1.74E-06 | 5.70E-04 |
| ENSG00000144645 | OSBPL10 | oxysterol binding protein like 10 | 0.25 | 1.78E-07 | 1.37E-04 |
| ENSG00000157657 | ZNF618 | zinc finger protein 618 | 0.27 | 2.95E-08 | 5.04E-05 |
| ENSG00000102962 | CCL22 | C-C motif chemokine ligand 22 | 0.27 | 1.72E-06 | 5.70E-04 |
| ENSG00000141404 | GNAL | G protein subunit alpha L | 0.31 | 1.62E-06 | 5.70E-04 |
| ENSG00000177311 | ZBTB38 | zinc finger and BTB domain containing 38 | 0.33 | 1.65E-06 | 5.70E-04 |
| ENSG00000100116 | GCAT | glycine C-acetyltransferase | 0.33 | 8.35E-06 | 1.66E-03 |
| ENSG00000160712 | IL6R | interleukin 6 receptor | 0.34 | 1.61E-06 | 5.70E-04 |
| ENSG00000198744 | MTCO3P12 | MT-CO3 pseudogene 12 | 0.37 | 1.30E-07 | 1.13E-04 |
| ENSG00000102317 | RBM3 | RNA binding motif protein 3 | 0.41 | 1.50E-07 | 1.23E-04 |
| ENSG00000229344 | MTCO2P12 | MT-CO2 pseudogene 12 | 0.42 | 2.48E-06 | 7.18E-04 |
| ENSG00000014257 | ACP3 | acid phosphatase 3 | 0.42 | 7.23E-07 | 3.34E-04 |
| ENSG00000110492 | MDK | midkine | 0.46 | 4.96E-06 | 1.15E-03 |
| ENSG00000203709 | MIR29B2CHG | MIR29B2 and MIR29C host gene | 0.47 | 8.12E-06 | 1.66E-03 |
| ENSG00000068724 | TTC7A | tetratricopeptide repeat domain 7A | 0.47 | 4.72E-06 | 1.13E-03 |
| ENSG00000105953 | OGDH | oxoglutarate dehydrogenase | 0.47 | 2.20E-06 | 6.73E-04 |
| ENSG00000160193 | WDR4 | WD repeat domain 4 | 0.48 | 9.76E-06 | 1.85E-03 |
| ENSG00000172053 | QARS1 | glutaminyl-tRNA synthetase 1 | 0.49 | 4.20E-06 | 1.10E-03 |
| ENSG00000005022 | SLC25A5 | solute carrier family 25 member 5 | 0.49 | 2.86E-06 | 7.94E-04 |

**Supplemental Table 7.** *Transcripts upregulated in HA Ramos cells compared to the normoxic controls (highlighted in grey are proteins upregulated in both HA cell lines) with fold change > 2 and P value < 0.00001.*

| **Ensembl** | **Gene Name** | **Description** | **Fold change** | **P value** | **FDR** |
| --- | --- | --- | --- | --- | --- |
| ENSG00000266976 | LOC102724908 | novel transcript | 166.36 | 1.81E-14 | 2.51E-10 |
| ENSG00000124788 | ATXN1 | ataxin 1 | 63.50 | 4.13E-08 | 2.12E-05 |
| ENSG00000162407 | PLPP3 | phospholipid phosphatase 3 | 20.35 | 3.12E-07 | 8.17E-05 |
| ENSG00000149972 | CNTN5 | contactin 5 | 18.99 | 7.23E-06 | 7.32E-04 |
| ENSG00000026751 | SLAMF7 | SLAM family member 7 | 14.64 | 2.36E-11 | 1.13E-07 |
| ENSG00000166897 | ELFN2 | extracellular leucine rich repeat and fibronectin type III domain containing 2 | 13.17 | 8.23E-08 | 3.26E-05 |
| ENSG00000120738 | EGR1 | early growth response 1 | 10.72 | 4.30E-06 | 5.19E-04 |
| ENSG00000109819 | PPARGC1A | PPARG coactivator 1 alpha | 10.36 | 2.09E-08 | 1.21E-05 |
| ENSG00000202198 |  | 7SK RNA | 10.11 | 9.91E-06 | 9.23E-04 |
| ENSG00000073282 | TP63 | tumor protein p63 | 9.62 | 5.06E-10 | 7.80E-07 |
| ENSG00000137752 | CASP1 | caspase 1 | 8.67 | 1.11E-09 | 1.31E-06 |
| ENSG00000143847 | PPFIA4 | PTPRF interacting protein alpha 4 | 8.53 | 2.42E-06 | 3.23E-04 |
| ENSG00000124785 | NRN1 | neuritin 1 | 8.18 | 2.11E-10 | 4.19E-07 |
| ENSG00000107957 | SH3PXD2A | SH3 and PX domains 2A | 7.83 | 3.30E-09 | 3.05E-06 |
| ENSG00000141526 | SLC16A3 | solute carrier family 16 member 3 | 7.34 | 6.16E-09 | 5.34E-06 |
| ENSG00000271553 |  | novel transcript | 7.06 | 1.13E-06 | 1.91E-04 |
| ENSG00000167208 | SNX20 | sorting nexin 20 | 6.91 | 2.30E-08 | 1.28E-05 |
| ENSG00000147180 | ZNF711 | zinc finger protein 711 | 6.36 | 2.60E-07 | 6.92E-05 |
| ENSG00000227028 | SLC8A1-AS1 | SLC8A1 antisense RNA 1 | 6.13 | 1.67E-07 | 5.25E-05 |
| ENSG00000204397 | CARD16 | caspase recruitment domain family member 16 | 6.13 | 8.42E-07 | 1.58E-04 |
| ENSG00000188277 | C15orf62 | chromosome 15 open reading frame 62 | 5.83 | 3.53E-07 | 8.61E-05 |
| ENSG00000224892 | RPS4XP16 | ribosomal protein S4X pseudogene 16 | 5.67 | 1.14E-06 | 1.91E-04 |
| ENSG00000049449 | RCN1 | reticulocalbin 1 | 5.55 | 2.67E-10 | 4.63E-07 |
| ENSG00000171189 | GRIK1 | glutamate ionotropic receptor kainate type subunit 1 | 5.51 | 3.30E-07 | 8.47E-05 |
| ENSG00000188404 | SELL | selectin L | 5.49 | 5.59E-08 | 2.50E-05 |
| ENSG00000211829 | TRDC | T cell receptor delta constant | 5.42 | 7.07E-09 | 5.76E-06 |
| ENSG00000112137 | PHACTR1 | phosphatase and actin regulator 1 | 5.24 | 2.44E-11 | 1.13E-07 |
| ENSG00000197358 | BNIP3P1 | BCL2 interacting protein 3 pseudogene 1 | 5.22 | 9.15E-09 | 7.05E-06 |
| ENSG00000139597 | N4BP2L1 | NEDD4 binding protein 2 like 1 | 5.22 | 1.31E-06 | 2.09E-04 |
| ENSG00000070371 | CLTCL1 | clathrin heavy chain like 1 | 5.05 | 3.69E-06 | 4.57E-04 |
| ENSG00000168685 | IL7R | interleukin 7 receptor | 4.92 | 1.07E-10 | 2.98E-07 |
| ENSG00000114268 | PFKFB4 | 6-phosphofructo-2-kinase/fructose-2,6-biphosphatase 4 | 4.59 | 8.12E-07 | 1.56E-04 |
| ENSG00000148926 | ADM | adrenomedullin | 4.29 | 1.14E-09 | 1.31E-06 |
| ENSG00000153253 | SCN3A | sodium voltage-gated channel alpha subunit 3 | 4.28 | 8.44E-10 | 1.17E-06 |
| ENSG00000168824 | NSG1 | neuronal vesicle trafficking associated 1 | 4.28 | 7.42E-06 | 7.46E-04 |
| ENSG00000016391 | CHDH | choline dehydrogenase | 4.07 | 6.85E-06 | 7.03E-04 |
| ENSG00000228451 | SDAD1P1 | SDA1 domain containing 1 pseudogene 1 | 4.07 | 6.37E-06 | 6.83E-04 |
| ENSG00000196954 | CASP4 | caspase 4 | 4.04 | 2.08E-10 | 4.19E-07 |
| ENSG00000136929 | HEMGN | hemogen | 3.97 | 4.79E-07 | 1.11E-04 |
| ENSG00000165966 | PDZRN4 | PDZ domain containing ring finger 4 | 3.88 | 8.99E-07 | 1.64E-04 |
| ENSG00000251002 | TRD-AS1 | TRD antisense RNA 1 | 3.83 | 2.26E-07 | 6.40E-05 |
| ENSG00000164938 | TP53INP1 | tumor protein p53 inducible nuclear protein 1 | 3.83 | 1.32E-08 | 9.49E-06 |
| ENSG00000180712 | LINC02363 | long intergenic non-protein coding RNA 2363 | 3.83 | 4.36E-08 | 2.16E-05 |
| ENSG00000121440 | PDZRN3 | PDZ domain containing ring finger 3 | 3.82 | 6.60E-06 | 6.83E-04 |
| ENSG00000272416 |  | novel transcript | 3.80 | 9.76E-07 | 1.74E-04 |
| ENSG00000267858 | MZF1-AS1 | MZF1 antisense RNA 1 | 3.79 | 2.03E-06 | 2.93E-04 |
| ENSG00000152503 | TRIM36 | tripartite motif containing 36 | 3.74 | 1.64E-06 | 2.45E-04 |
| ENSG00000197043 | ANXA6 | annexin A6 | 3.66 | 3.04E-09 | 3.02E-06 |
| ENSG00000231890 | DARS1-AS1 | DARS1 antisense RNA 1 | 3.66 | 4.44E-07 | 1.04E-04 |
| ENSG00000108797 | CNTNAP1 | contactin associated protein 1 | 3.64 | 7.87E-07 | 1.55E-04 |
| ENSG00000206530 | CFAP44 | cilia and flagella associated protein 44 | 3.63 | 1.38E-07 | 4.57E-05 |
| ENSG00000119042 | SATB2 | SATB homeobox 2 | 3.63 | 9.54E-08 | 3.58E-05 |
| ENSG00000162433 | AK4 | adenylate kinase 4 | 3.61 | 3.99E-07 | 9.55E-05 |
| ENSG00000122884 | P4HA1 | prolyl 4-hydroxylase subunit alpha 1 | 3.59 | 1.63E-09 | 1.74E-06 |
| ENSG00000227507 | LTB | lymphotoxin beta | 3.47 | 2.34E-07 | 6.41E-05 |
| ENSG00000180113 | TDRD6 | tudor domain containing 6 | 3.47 | 2.40E-06 | 3.23E-04 |
| ENSG00000230266 | XXYLT1-AS2 | XXYLT1 antisense RNA 2 | 3.42 | 1.31E-06 | 2.09E-04 |
| ENSG00000064225 | ST3GAL6 | ST3 beta-galactoside alpha-2,3-sialyltransferase 6 | 3.40 | 7.47E-08 | 3.04E-05 |
| ENSG00000135925 | WNT10A | Wnt family member 10A | 3.37 | 6.43E-08 | 2.70E-05 |
| ENSG00000118515 | SGK1 | serum/glucocorticoid regulated kinase 1 | 3.37 | 6.52E-06 | 6.83E-04 |
| ENSG00000136848 | DAB2IP | DAB2 interacting protein | 3.30 | 4.62E-08 | 2.21E-05 |
| ENSG00000127124 | HIVEP3 | HIVEP zinc finger 3 | 3.28 | 1.73E-08 | 1.04E-05 |
| ENSG00000125089 | SH3TC1 | SH3 domain and tetratricopeptide repeats 1 | 3.23 | 1.34E-07 | 4.53E-05 |
| ENSG00000255031 |  | novel transcript, antisense to CHKA | 3.23 | 2.21E-06 | 3.09E-04 |
| ENSG00000176171 | BNIP3 | BCL2 interacting protein 3 | 3.23 | 1.04E-07 | 3.80E-05 |
| ENSG00000253686 | LINC01484 | long intergenic non-protein coding RNA 1484 | 3.22 | 3.09E-06 | 4.04E-04 |
| ENSG00000107742 | SPOCK2 | SPARC (osteonectin), cwcv and kazal like domains proteoglycan 2 | 3.15 | 2.05E-06 | 2.93E-04 |
| ENSG00000117090 | SLAMF1 | signaling lymphocytic activation molecule family member 1 | 3.09 | 1.40E-06 | 2.18E-04 |
| ENSG00000002586 | CD99 | CD99 molecule (Xg blood group) | 3.07 | 1.74E-07 | 5.36E-05 |
| ENSG00000110400 | NECTIN1 | nectin cell adhesion molecule 1 | 3.06 | 1.89E-07 | 5.68E-05 |
| ENSG00000120279 | MYCT1 | MYC target 1 | 3.06 | 1.29E-07 | 4.52E-05 |
| ENSG00000147168 | IL2RG | interleukin 2 receptor subunit gamma | 3.04 | 1.59E-08 | 1.00E-05 |
| ENSG00000179873 | NLRP11 | NLR family pyrin domain containing 11 | 2.95 | 2.22E-07 | 6.40E-05 |
| ENSG00000183508 | TENT5C | terminal nucleotidyltransferase 5C | 2.90 | 4.40E-06 | 5.22E-04 |
| ENSG00000110934 | BIN2 | bridging integrator 2 | 2.89 | 5.37E-07 | 1.20E-04 |
| ENSG00000100628 | ASB2 | ankyrin repeat and SOCS box containing 2 | 2.88 | 5.67E-07 | 1.25E-04 |
| ENSG00000164849 | GPR146 | G protein-coupled receptor 146 | 2.82 | 5.53E-06 | 6.18E-04 |
| ENSG00000178163 | ZNF518B | zinc finger protein 518B | 2.81 | 5.59E-06 | 6.20E-04 |
| ENSG00000083444 | PLOD1 | procollagen-lysine,2-oxoglutarate 5-dioxygenase 1 | 2.80 | 5.16E-08 | 2.38E-05 |
| ENSG00000180998 | GPR137C | G protein-coupled receptor 137C | 2.79 | 1.83E-06 | 2.70E-04 |
| ENSG00000177409 | SAMD9L | sterile alpha motif domain containing 9 like | 2.74 | 6.64E-07 | 1.37E-04 |
| ENSG00000197279 | ZNF165 | zinc finger protein 165 | 2.73 | 7.20E-06 | 7.32E-04 |
| ENSG00000129993 | CBFA2T3 | CBFA2/RUNX1 partner transcriptional co-repressor 3 | 2.70 | 6.15E-07 | 1.31E-04 |
| ENSG00000104765 | BNIP3L | BCL2 interacting protein 3 like | 2.70 | 1.53E-07 | 4.93E-05 |
| ENSG00000167634 | NLRP7 | NLR family pyrin domain containing 7 | 2.68 | 2.24E-06 | 3.10E-04 |
| ENSG00000130592 | LSP1 | lymphocyte specific protein 1 | 2.67 | 1.31E-07 | 4.52E-05 |
| ENSG00000009790 | TRAF3IP3 | TRAF3 interacting protein 3 | 2.65 | 3.50E-07 | 8.61E-05 |
| ENSG00000171867 | PRNP | prion protein (Kanno blood group) | 2.59 | 9.37E-06 | 8.90E-04 |
| ENSG00000156675 | RAB11FIP1 | RAB11 family interacting protein 1 | 2.59 | 4.47E-06 | 5.25E-04 |
| ENSG00000164543 | STK17A | serine/threonine kinase 17a | 2.51 | 5.37E-07 | 1.20E-04 |
| ENSG00000230606 | APPAT | atherosclerotic plaque pathogenesis associated transcript | 2.48 | 6.23E-07 | 1.31E-04 |
| ENSG00000123609 | NMI | N-myc and STAT interactor | 2.47 | 2.41E-06 | 3.23E-04 |
| ENSG00000184588 | PDE4B | phosphodiesterase 4B | 2.46 | 1.56E-06 | 2.36E-04 |
| ENSG00000118503 | TNFAIP3 | TNF alpha induced protein 3 | 2.44 | 3.66E-06 | 4.57E-04 |
| ENSG00000112715 | VEGFA | vascular endothelial growth factor A | 2.41 | 8.36E-07 | 1.58E-04 |
| ENSG00000173530 | TNFRSF10D | TNF receptor superfamily member 10d | 2.40 | 6.17E-06 | 6.68E-04 |
| ENSG00000120688 | WBP4 | WW domain binding protein 4 | 2.39 | 3.54E-07 | 8.61E-05 |
| ENSG00000175265 | GOLGA8A | golgin A8 family member A | 2.38 | 3.79E-06 | 4.65E-04 |
| ENSG00000196968 | FUT11 | fucosyltransferase 11 | 2.38 | 9.66E-07 | 1.74E-04 |
| ENSG00000146278 | PNRC1 | proline rich nuclear receptor coactivator 1 | 2.35 | 6.72E-07 | 1.37E-04 |
| ENSG00000113742 | CPEB4 | cytoplasmic polyadenylation element binding protein 4 | 2.35 | 4.32E-06 | 5.19E-04 |
| ENSG00000121060 | TRIM25 | tripartite motif containing 25 | 2.34 | 2.36E-06 | 3.23E-04 |
| ENSG00000145730 | PAM | peptidylglycine alpha-amidating monooxygenase | 2.34 | 9.72E-06 | 9.17E-04 |
| ENSG00000182013 | PNMA8A | PNMA family member 8A | 2.32 | 5.38E-06 | 6.07E-04 |
| ENSG00000229164 | TRAC | T cell receptor alpha constant | 2.29 | 1.05E-06 | 1.83E-04 |
| ENSG00000245614 | DDX11-AS1 | DDX11 antisense RNA 1 | 2.29 | 1.13E-06 | 1.91E-04 |
| ENSG00000071575 | TRIB2 | tribbles pseudokinase 2 | 2.24 | 1.92E-06 | 2.81E-04 |
| ENSG00000118432 | CNR1 | cannabinoid receptor 1 | 2.22 | 2.36E-07 | 6.41E-05 |
| ENSG00000115738 | ID2 | inhibitor of DNA binding 2 | 2.17 | 1.20E-06 | 1.97E-04 |
| ENSG00000145348 | TBCK | TBC1 domain containing kinase | 2.15 | 4.92E-06 | 5.64E-04 |
| ENSG00000151575 | TEX9 | testis expressed 9 | 2.13 | 5.76E-06 | 6.34E-04 |
| ENSG00000136870 | ZNF189 | zinc finger protein 189 | 2.08 | 5.31E-06 | 6.03E-04 |
| ENSG00000172086 | KRCC1 | lysine rich coiled-coil 1 | 2.07 | 5.94E-06 | 6.48E-04 |
| ENSG00000160213 | CSTB | cystatin B | 2.07 | 8.41E-06 | 8.27E-04 |
| ENSG00000134508 | CABLES1 | Cdk5 and Abl enzyme substrate 1 | 2.06 | 3.24E-06 | 4.20E-04 |
| ENSG00000134986 | NREP | neuronal regeneration related protein | 2.03 | 3.63E-06 | 4.57E-04 |
| ENSG00000115419 | GLS | glutaminase | 2.03 | 8.75E-06 | 8.48E-04 |

**Supplemental Table 8.** *Transcripts downregulated in HA Ramos cells compared to the normoxic controls (highlighted in grey are proteins downregulated in both HA cell lines) with fold change < 0.5 and P value < 0.00001.*

| **Ensembl** | **Gene Name** | **Description** | **Fold change** | **P value** | **FDR** |
| --- | --- | --- | --- | --- | --- |
| ENSG00000163393 | SLC22A15 | solute carrier family 22 member 15 | 0.11 | 1.56E-06 | 2.36E-04 |
| ENSG00000130830 | MPP1 | MAGUK p55 scaffold protein 1 | 0.12 | 6.16E-07 | 1.31E-04 |
| ENSG00000237940 | LINC01238 | Long Intergenic Non-Protein Coding RNA 1238 | 0.15 | 9.24E-08 | 3.56E-05 |
| ENSG00000103021 | CFAP263 | cilia and flagella associated protein 263 | 0.15 | 4.87E-06 | 5.62E-04 |
| ENSG00000079102 | RUNX1T1 | RUNX1 partner transcriptional co-repressor 1 | 0.19 | 2.01E-07 | 5.92E-05 |
| ENSG00000061918 | GUCY1B1 | guanylate cyclase 1 soluble subunit beta 1 | 0.20 | 1.36E-06 | 2.14E-04 |
| ENSG00000155755 | TMEM237 | transmembrane protein 237 | 0.22 | 2.51E-06 | 3.32E-04 |
| ENSG00000114948 | ADAM23 | ADAM metallopeptidase domain 23 | 0.23 | 6.43E-11 | 2.23E-07 |
| ENSG00000136040 | PLXNC1 | plexin C1 | 0.25 | 1.37E-08 | 9.49E-06 |
| ENSG00000144199 | FAHD2B | fumarylacetoacetate hydrolase domain containing 2B | 0.26 | 4.34E-06 | 5.19E-04 |
| ENSG00000119547 | ONECUT2 | one cut homeobox 2 | 0.27 | 6.59E-06 | 6.83E-04 |
| ENSG00000186710 | CFAP73 | cilia and flagella associated protein 73 | 0.28 | 6.54E-06 | 6.83E-04 |
| ENSG00000173320 | STOX2 | storkhead box 2 | 0.28 | 2.54E-08 | 1.35E-05 |
| ENSG00000058668 | ATP2B4 | ATPase plasma membrane Ca2+ transporting 4 | 0.29 | 1.48E-08 | 9.75E-06 |
| ENSG00000110042 | DTX4 | deltex E3 ubiquitin ligase 4 | 0.30 | 8.53E-07 | 1.58E-04 |
| ENSG00000172264 | MACROD2 | mono-ADP ribosylhydrolase 2 | 0.30 | 7.92E-07 | 1.55E-04 |
| ENSG00000104154 | SLC30A4 | solute carrier family 30 member 4 | 0.37 | 7.12E-07 | 1.43E-04 |
| ENSG00000159445 | THEM4 | thioesterase superfamily member 4 | 0.37 | 1.41E-06 | 2.18E-04 |
| ENSG00000102317 | RBM3 | RNA binding motif protein 3 | 0.39 | 6.05E-08 | 2.62E-05 |
| ENSG00000082438 | COBLL1 | cordon-bleu WH2 repeat protein like 1 | 0.41 | 7.96E-06 | 7.94E-04 |
| ENSG00000213903 | LTB4R | leukotriene B4 receptor | 0.43 | 2.09E-06 | 2.96E-04 |
| ENSG00000135643 | KCNMB4 | potassium calcium-activated channel subfamily M regulatory beta subunit 4 | 0.44 | 4.53E-06 | 5.28E-04 |
| ENSG00000132846 | ZBED3 | zinc finger BED-type containing 3 | 0.45 | 6.41E-06 | 6.83E-04 |
| ENSG00000127325 | BEST3 | bestrophin 3 | 0.46 | 1.21E-06 | 1.97E-04 |
| ENSG00000112182 | BACH2 | BTB domain and CNC homolog 2 | 0.48 | 1.01E-06 | 1.77E-04 |
| ENSG00000136877 | FPGS | folylpolyglutamate synthase | 0.49 | 8.73E-06 | 8.48E-04 |
| ENSG00000183207 | RUVBL2 | RuvB like AAA ATPase 2 | 0.49 | 8.19E-06 | 8.11E-04 |
| ENSG00000169100 | SLC25A6 | solute carrier family 25 member 6 | 0.50 | 9.91E-06 | 9.23E-04 |

**Supplemental Table 9.** *Proteins significantly upregulated in HA HBL2 cells compared to the normoxic controls (highlighted in grey are proteins upregulated in both HA cell lines)*

| **Entry** | **Gene Name** | **Description** | **Unique Peptides** | **Fold-change** | **Adj. P-Value** |
| --- | --- | --- | --- | --- | --- |
| Q01432 | AMPD3 | AMP deaminase 3 | 7 | 3.00 | 0.0054 |
| Q9H9S4 | CAB39L | Calcium-binding protein 39-like | 2 | 3.00 | 0.0354 |
| P06239 | LCK | Tyrosine-protein kinase Lck | 15 | 2.99 | 0.0396 |
| P13674 | P4HA1 | Prolyl 4-hydroxylase subunit alpha-1 | 29 | 2.15 | 0.0295 |
| Q969X5 | ERGIC1 | Endoplasmic reticulum-Golgi intermediate compartment protein 1 | 8 | 2.04 | 0.0450 |
| Q16566 | CAMK4 | Calcium/calmodulin-dependent protein kinase type IV | 8 | 1.98 | 0.0022 |
| P09104 | ENO2 | Gamma-enolase | 10 | 1.89 | 0.0434 |
| Q6ZSZ5 | ARHGEF18 | Rho guanine nucleotide exchange factor 18 | 14 | 1.81 | 0.0085 |
| Q16555 | DPYSL2 | Dihydropyrimidinase-related protein 2 | 25 | 1.76 | 0.0100 |
| Q9BT09 | CNPY3 | Protein canopy homolog 3 | 12 | 1.75 | 0.0086 |
| P82970 | HMGN5 | High mobility group nucleosome-binding domain-containing protein 5 | 6 | 1.74 | 0.0354 |
| Q9NUQ2 | AGPAT5 | 1-acyl-sn-glycerol-3-phosphate acyltransferase epsilon | 5 | 1.72 | 0.0160 |
| P10114 | RAP2A | Ras-related protein Rap-2a | 3 | 1.70 | 0.0050 |
| Q14534 | SQLE | Squalene monooxygenase | 20 | 1.63 | 0.0119 |
| P18669 | PGAM1 | Phosphoglycerate mutase 1 | 11 | 1.58 | 0.0022 |
| Q9NZ45 | CISD1 | CDGSH iron-sulfur domain-containing protein 1 | 4 | 1.58 | 0.0037 |
| Q13509 | TUBB3 | Tubulin beta-3 chain | 6 | 1.55 | 0.0331 |
| Q9UHD9 | UBQLN2 | Ubiquilin-2 | 4 | 1.54 | 0.0329 |
| Q9Y2D5 | AKAP2 | A-kinase anchor protein 2 | 17 | 1.54 | 0.0431 |
| P48651 | PTDSS1 | Phosphatidylserine synthase 1 | 7 | 1.52 | 0.0331 |

**Supplemental Table 10.** *Proteins significantly downregulated in HA HBL2 cells compared to the normoxic controls (highlighted in grey are proteins downregulated in both HA cell lines)*

| **Entry** | **Gene Name** | **Description** | **Unique Peptides** | **Fold-change** | **Adj. P-Value** |
| --- | --- | --- | --- | --- | --- |
| Q9Y6M9 | NDUFB9 | NADH dehydrogenase [ubiquinone] 1 beta subcomplex subunit 9 | 5 | 0.34 | 0.0479 |
| Q4U2R6 | MRPL51 | 39S ribosomal protein L51, mitochondrial | 2 | 0.37 | 0.0022 |
| A4D1E9 | GTPBP10 | GTP-binding protein 10 | 3 | 0.39 | 0.0345 |
| O95299 | NDUFA10 | NADH dehydrogenase [ubiquinone] 1 alpha subcomplex subunit 10, mitochondrial | 8 | 0.41 | 0.0242 |
| Q13084 | MRPL28 | 39S ribosomal protein L28, mitochondrial | 6 | 0.42 | 0.0282 |
| Q9Y3B7 | MRPL11 | 39S ribosomal protein L11, mitochondrial | 9 | 0.42 | 0.0354 |
| O75438 | NDUFB1 | NADH dehydrogenase [ubiquinone] 1 beta subcomplex subunit 1 | 2 | 0.43 | 0.0076 |
| Q86Y39 | NDUFA11 | NADH dehydrogenase [ubiquinone] 1 alpha subcomplex subunit 11 | 3 | 0.43 | 0.0054 |
| Q9BRJ2 | MRPL45 | 39S ribosomal protein L45, mitochondrial | 4 | 0.43 | 0.0354 |
| Q9BYD3 | MRPL4 | 39S ribosomal protein L4, mitochondrial | 8 | 0.43 | 0.0370 |
| Q9UI09 | NDUFA12 | NADH dehydrogenase [ubiquinone] 1 alpha subcomplex subunit 12 | 3 | 0.43 | 0.0354 |
| P49406 | MRPL19 | 39S ribosomal protein L19, mitochondrial | 7 | 0.43 | 0.0100 |
| Q9Y2R9 | MRPS7 | 28S ribosomal protein S7, mitochondrial | 6 | 0.43 | 0.0354 |
| P09669 | COX6C | Cytochrome c oxidase subunit 6C | 4 | 0.44 | 0.0246 |
| P17568 | NDUFB7 | NADH dehydrogenase [ubiquinone] 1 beta subcomplex subunit 7 | 5 | 0.44 | 0.0492 |
| O75380 | NDUFS6 | NADH dehydrogenase [ubiquinone] iron-sulfur protein 6, mitochondrial | 4 | 0.46 | 0.0354 |
| Q8NDA8 | MROH1 | Maestro heat-like repeat-containing protein family member 1 | 2 | 0.47 | 0.0455 |
| O75616 | ERAL1 | GTPase Era, mitochondrial | 4 | 0.47 | 0.0425 |
| Q9NQ50 | MRPL40 | 39S ribosomal protein L40, mitochondrial | 5 | 0.47 | 0.0100 |
| P51398 | DAP3 | 28S ribosomal protein S29, mitochondrial | 14 | 0.47 | 0.0354 |
| Q9Y2V7 | COG6 | Conserved oligomeric Golgi complex subunit 6 | 5 | 0.47 | 0.0450 |
| Q96GC5 | MRPL48 | 39S ribosomal protein L48, mitochondrial | 2 | 0.48 | 0.0331 |
| Q96DV4 | MRPL38 | 39S ribosomal protein L38, mitochondrial | 3 | 0.48 | 0.0455 |
| Q9GZT3 | SLIRP | SRA stem-loop-interacting RNA-binding protein, mitochondrial | 9 | 0.49 | 0.0331 |
| P13284 | IFI30 | Gamma-interferon-inducible lysosomal thiol reductase | 3 | 0.49 | 0.0367 |
| Q9NYY8 | FASTKD2 | FAST kinase domain-containing protein 2, mitochondrial | 13 | 0.49 | 0.0443 |
| P15954 | COX7C | Cytochrome c oxidase subunit 7C, mitochondrial | 3 | 0.50 | 0.0458 |
| Q9H9J2 | MRPL44 | 39S ribosomal protein L44, mitochondrial | 8 | 0.50 | 0.0160 |
| P82673 | MRPS35 | 28S ribosomal protein S35, mitochondrial | 5 | 0.51 | 0.0345 |
| Q9HD33 | MRPL47 | 39S ribosomal protein L47, mitochondrial | 7 | 0.52 | 0.0478 |
| Q9BRP1 | PDCD2L | Programmed cell death protein 2-like | 3 | 0.52 | 0.0192 |
| P42704 | LRPPRC | Leucine-rich PPR motif-containing protein, mitochondrial | 89 | 0.52 | 0.0354 |
| Q9NYK5 | MRPL39 | 39S ribosomal protein L39, mitochondrial | 10 | 0.53 | 0.0143 |
| O95644 | NFATC1 | Nuclear factor of activated T-cells, cytoplasmic 1 | 2 | 0.53 | 0.0455 |
| Q13405 | MRPL49 | 39S ribosomal protein L49, mitochondrial | 5 | 0.54 | 0.0354 |
| P82675 | MRPS5 | 28S ribosomal protein S5, mitochondrial | 9 | 0.54 | 0.0434 |
| Q16795 | NDUFA9 | NADH dehydrogenase [ubiquinone] 1 alpha subcomplex subunit 9, mitochondrial | 13 | 0.54 | 0.0492 |
| Q7L2E3 | DHX30 | ATP-dependent RNA helicase DHX30 | 32 | 0.55 | 0.0381 |
| Q92552 | MRPS27 | 28S ribosomal protein S27, mitochondrial | 12 | 0.56 | 0.0354 |
| P82914 | MRPS15 | 28S ribosomal protein S15, mitochondrial | 7 | 0.56 | 0.0354 |
| Q7Z7H8 | MRPL10 | 39S ribosomal protein L10, mitochondrial | 6 | 0.57 | 0.0035 |
| Q9BZE1 | MRPL37 | 39S ribosomal protein L37, mitochondrial | 10 | 0.58 | 0.0354 |
| P19404 | NDUFV2 | NADH dehydrogenase [ubiquinone] flavoprotein 2, mitochondrial | 11 | 0.59 | 0.0345 |
| Q9NP92 | MRPS30 | 39S ribosomal protein S30, mitochondrial | 5 | 0.60 | 0.0354 |
| Q9BQC6 | MRPL57 | Ribosomal protein 63, mitochondrial | 2 | 0.63 | 0.0421 |
| P82932 | MRPS6 | 28S ribosomal protein S6, mitochondrial | 5 | 0.64 | 0.0458 |
| P36969 | GPX4 | Phospholipid hydroperoxide glutathione peroxidase | 6 | 0.64 | 0.0400 |
| O43837 | IDH3B | Isocitrate dehydrogenase [NAD] subunit beta, mitochondrial | 11 | 0.65 | 0.0434 |

**Supplemental Table 11.** *Proteins significantly upregulated in HA Ramos cells compared to the normoxic controls (highlighted in grey are proteins upregulated in both HA cell lines)*

| **Entry** | **Gene Name** | **Description** | **Unique Peptides** | **Fold-change** | **Adj. P-Value** |
| --- | --- | --- | --- | --- | --- |
| O15427 | SLC16A3 | Monocarboxylate transporter 4 | 4 | 3.62 | 0.0097 |
| P29466 | CASP1 | Caspase-1 | 7 | 3.47 | 0.0213 |
| P02792 | FTL | Ferritin light chain | 3 | 3.46 | 0.0455 |
| P32970 | CD70 | CD70 antigen | 4 | 2.93 | 0.0034 |
| O00584 | RNASET2 | Ribonuclease T2 | 6 | 2.88 | 0.0012 |
| Q9NZ45 | CISD1 | CDGSH iron-sulfur domain-containing protein 1 | 4 | 2.82 | 0.0000 |
| P51648 | ALDH3A2 | Aldehyde dehydrogenase family 3 member A2 | 3 | 2.63 | 0.0046 |
| P13674 | P4HA1 | Prolyl 4-hydroxylase subunit alpha-1 | 29 | 2.49 | 0.0064 |
| P50416 | CPT1A | Carnitine O-palmitoyltransferase 1, liver isoform | 17 | 2.44 | 0.0047 |
| P49662 | CASP4 | Caspase-4 | 8 | 2.41 | 0.0217 |
| Q9H8Y5 | ANKZF1 | Ankyrin repeat and zinc finger domain-containing protein 1 | 7 | 2.33 | 0.0064 |
| P09972 | ALDOC | Fructose-bisphosphate aldolase C | 18 | 2.31 | 0.0096 |
| P04080 | CSTB | Cystatin-B | 6 | 2.30 | 0.0170 |
| Q9C0E8 | LNPK | Endoplasmic reticulum junction formation protein lunapark | 4 | 2.21 | 0.0230 |
| Q14573 | ITPR3 | Inositol 1,4,5-trisphosphate receptor type 3 | 12 | 2.15 | 0.0217 |
| Q96A26 | FAM162A | Protein FAM162A | 7 | 2.12 | 0.0000 |
| O15031 | PLXNB2 | Plexin-B2 | 18 | 2.08 | 0.0010 |
| Q16850 | CYP51A1 | Lanosterol 14-alpha demethylase | 14 | 2.03 | 0.0109 |
| Q4G176 | ACSF3 | Malonate--CoA ligase ACSF3, mitochondrial | 13 | 2.00 | 0.0144 |
| Q8N5K1 | CISD2 | CDGSH iron-sulfur domain-containing protein 2 | 6 | 1.95 | 0.0058 |
| P33241 | LSP1 | Lymphocyte-specific protein 1 | 16 | 1.93 | 0.0058 |
| Q9NRZ7 | AGPAT3 | 1-acyl-sn-glycerol-3-phosphate acyltransferase gamma | 4 | 1.86 | 0.0492 |
| P60174 | TPI1 | Triosephosphate isomerase | 20 | 1.84 | 0.0001 |
| Q14005 | IL16 | Pro-interleukin-16 | 16 | 1.83 | 0.0000 |
| P22732 | SLC2A5 | Solute carrier family 2, facilitated glucose transporter member 5 | 3 | 1.83 | 0.0236 |
| Q01432 | AMPD3 | AMP deaminase 3 | 7 | 1.82 | 0.0239 |
| Q9BQB6 | VKORC1 | Vitamin K epoxide reductase complex subunit 1 | 3 | 1.77 | 0.0237 |
| P19367 | HK1 | Hexokinase-1 | 20 | 1.75 | 0.0058 |
| P30040 | ERP29 | Endoplasmic reticulum resident protein 29 | 15 | 1.75 | 0.0088 |
| P62328 | TMSB4X | Thymosin beta-4 | 3 | 1.75 | 0.0119 |
| Q9UDX5 | MTFP1 | Mitochondrial fission process protein 1 | 6 | 1.71 | 0.0046 |
| Q9Y4C1 | KDM3A | Lysine-specific demethylase 3A | 14 | 1.71 | 0.0116 |
| Q9UDY2 | TJP2 | Tight junction protein ZO-2 | 19 | 1.71 | 0.0058 |
| P11166 | SLC2A1 | Solute carrier family 2, facilitated glucose transporter member 1 | 6 | 1.70 | 0.0116 |
| O75915 | ARL6IP5 | PRA1 family protein 3 | 5 | 1.67 | 0.0473 |
| Q9Y394 | DHRS7 | Dehydrogenase/reductase SDR family member 7 | 7 | 1.64 | 0.0205 |
| P18669 | PGAM1 | Phosphoglycerate mutase 1 | 11 | 1.64 | 0.0010 |
| P36551 | CPOX | Oxygen-dependent coproporphyrinogen-III oxidase, mitochondrial | 10 | 1.64 | 0.0205 |
| P06733 | ENO1 | Alpha-enolase | 32 | 1.64 | 0.0058 |
| Q12882 | DPYD | Dihydropyrimidine dehydrogenase [NADP(+)] | 4 | 1.62 | 0.0348 |
| P05556 | ITGB1 | Integrin beta-1 | 17 | 1.61 | 0.0060 |
| Q14320 | FAM50A | Protein FAM50A | 6 | 1.60 | 0.0077 |
| P07919 | UQCRH | Cytochrome b-c1 complex subunit 6, mitochondrial | 6 | 1.59 | 0.0127 |
| Q9GZT9 | EGLN1 | Egl nine homolog 1 | 13 | 1.58 | 0.0046 |
| O00522 | KRIT1 | Krev interaction trapped protein 1 | 3 | 1.57 | 0.0217 |
| P09211 | GSTP1 | Glutathione S-transferase P | 13 | 1.57 | 0.0088 |
| Q14534 | SQLE | Squalene monooxygenase | 20 | 1.57 | 0.0126 |
| Q96HE7 | ERO1A | ERO1-like protein alpha | 17 | 1.56 | 0.0224 |
| Q9BT09 | CNPY3 | Protein canopy homolog 3 | 12 | 1.56 | 0.0239 |
| Q9P016 | THYN1 | Thymocyte nuclear protein 1 | 14 | 1.54 | 0.0217 |
| Q6PIU2 | NCEH1 | Neutral cholesterol ester hydrolase 1 | 5 | 1.54 | 0.0239 |
| Q9ULE6 | PALD1 | Paladin | 13 | 1.54 | 0.0309 |
| Q9Y5Y6 | ST14 | Suppressor of tumorigenicity 14 protein | 8 | 1.54 | 0.0116 |
| P34949 | MPI | Mannose-6-phosphate isomerase | 7 | 1.53 | 0.0289 |
| Q96C19 | EFHD2 | EF-hand domain-containing protein D2 | 13 | 1.52 | 0.0098 |
| Q16762 | TST | Thiosulfate sulfurtransferase | 7 | 1.52 | 0.0237 |
| P14618 | PKM | Pyruvate kinase PKM | 46 | 1.51 | 0.0010 |
| O60256 | PRPSAP2 | Phosphoribosyl pyrophosphate synthase-associated protein 2 | 10 | 1.51 | 0.0067 |

**Supplemental Table 12.** *Proteins significantly downregulated in HA Ramos cells compared to the normoxic controls (highlighted in grey are proteins downregulated in both HA cell lines)*

| **Entry** | **Gene Name** | **Description** | **Unique Peptides** | **Fold-change** | **Adj. P-Value** |
| --- | --- | --- | --- | --- | --- |
| P43363 | MAGEA10 | Melanoma-associated antigen 10 | 4 | 0.22 | 0.0106 |
| P46821 | MAP1B | Microtubule-associated protein 1B | 16 | 0.32 | 0.0004 |
| P05166 | PCCB | Propionyl-CoA carboxylase beta chain, mitochondrial | 12 | 0.44 | 0.0301 |
| P21333 | FLNA | Filamin-A | 67 | 0.45 | 0.0000 |
| P78559 | MAP1A | Microtubule-associated protein 1A | 27 | 0.45 | 0.0044 |
| P07355 | ANXA2 | Annexin A2 | 27 | 0.45 | 0.0058 |
| O75616 | ERAL1 | GTPase Era, mitochondrial | 4 | 0.45 | 0.0217 |
| P09669 | COX6C | Cytochrome c oxidase subunit 6C | 4 | 0.47 | 0.0162 |
| P15954 | COX7C | Cytochrome c oxidase subunit 7C, mitochondrial | 3 | 0.47 | 0.0239 |
| P50579 | METAP2 | Methionine aminopeptidase 2 | 18 | 0.48 | 0.0058 |
| O75369 | FLNB | Filamin-B | 79 | 0.49 | 0.0000 |
| Q16643 | DBN1 | Drebrin | 18 | 0.53 | 0.0058 |
| P09622 | DLD | Dihydrolipoyl dehydrogenase, mitochondrial | 14 | 0.53 | 0.0239 |
| Q5T6V5 | QNG1 | Queuosine salvage protein | 2 | 0.54 | 0.0456 |
| P27105 | STOM | Stomatin | 6 | 0.55 | 0.0239 |
| Q96GM8 | TOE1 | Target of EGR1 protein 1 | 8 | 0.62 | 0.0435 |
| Q9BY32 | ITPA | Inosine triphosphate pyrophosphatase | 9 | 0.66 | 0.0435 |
