# Supplementary material for: Long-term adaptation of lymphoma cell lines to hypoxia is mediated by diverse molecular mechanisms that are targetable with specific inhibitors
Source: Cell Death Discov. 2025 Feb 18;11:65. doi: 10.1038/s41420-025-02341-y (PMC11836139; doi:10.1038/s41420-025-02341-y)
Supplement: Supplementary file 2 — Supplemental Western Blots [file 41420_2025_2341_MOESM2_ESM.docx]

**SUPPLEMENTAL WESTERN BLOTS**

***Long-term adaptation of lymphoma cell lines to hypoxia is mediated by diverse molecular mechanisms that are targetable with specific inhibitors***

**Running Title:** Adaptation of lymphomas to long-term hypoxia

**Authors:** Lenka Daumova^1^, Dmitry Manakov^1^, Jiri Petrak^2^, Dana Sovilj3, Matěj Behounek^2^, Ladislav Andera^3,4^, Ondrej Vit^2^, Olga Souckova^5^, Ondrej Havranek^2,6^, Nicol Renesova1, Alex Dolnikova^1^, Liliana Tuskova^1,6^, Lucie Winkovska^7^, Nardjas Bettazova^1,8^, Kristina Kupcova^2,6^, Marie HubalekKalbacova^1^, Miriama Sikorova^1^, Marek Trneny^6^, and Pavel Klener^1,6*^

**Corresponding author*

| **Target** | **kDa** | **Chemiluminescence** | **Colorimetric (Precision Plus Protein Standards #161-0374)** |
| --- | --- | --- | --- |
| Bcl-2 | 28 | 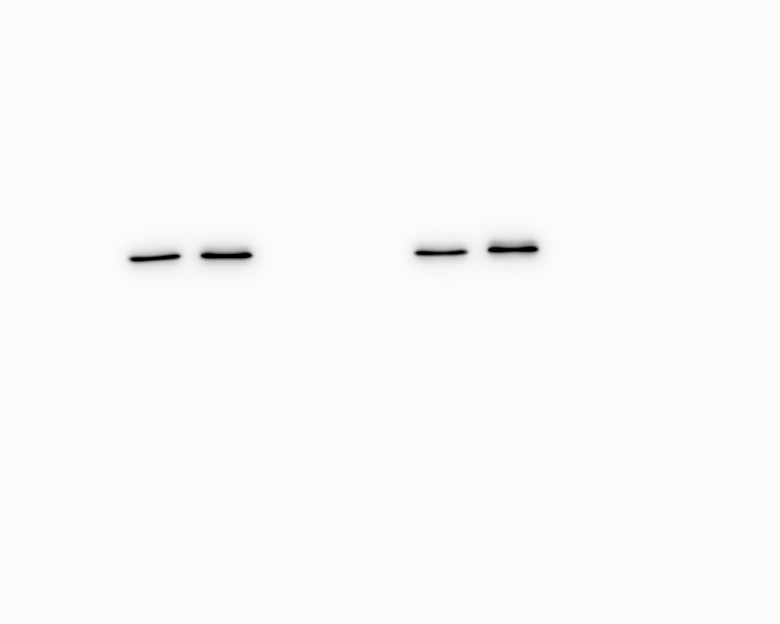 | 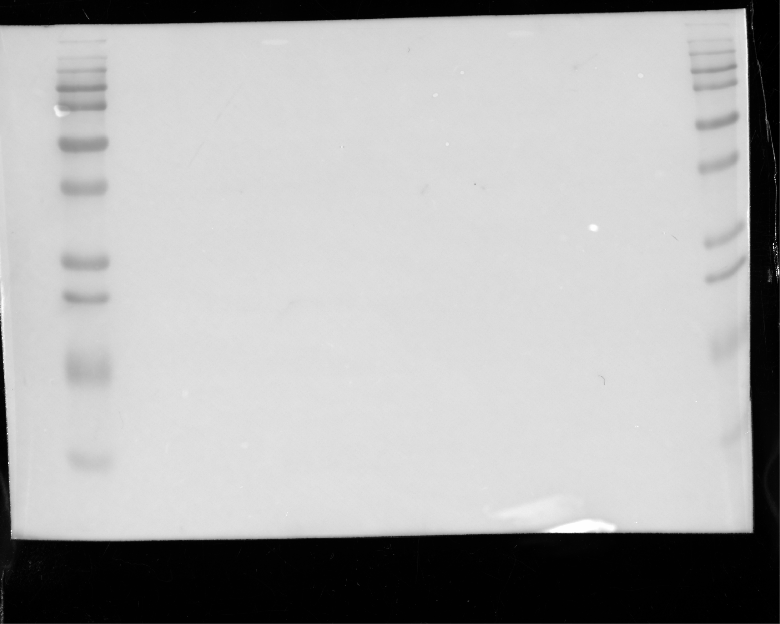 |
| α-tubulin to Bcl-2 | 50 | 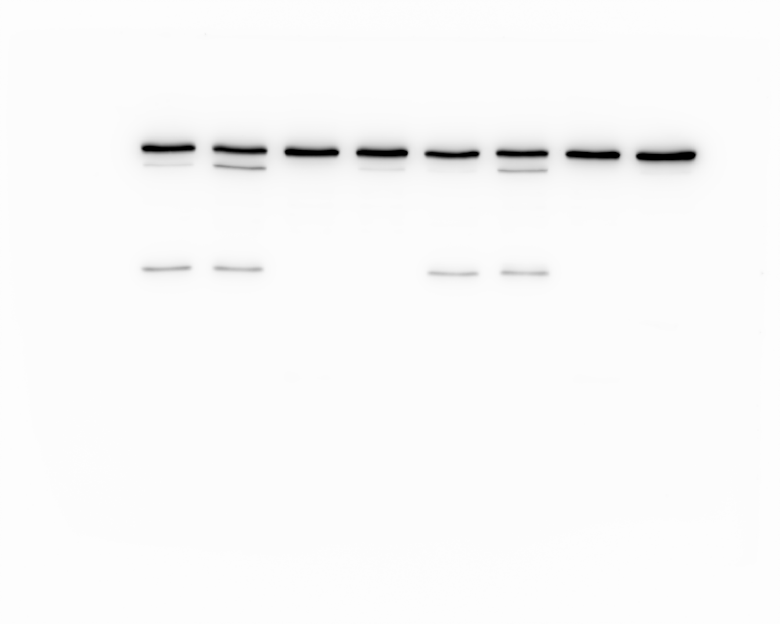 | 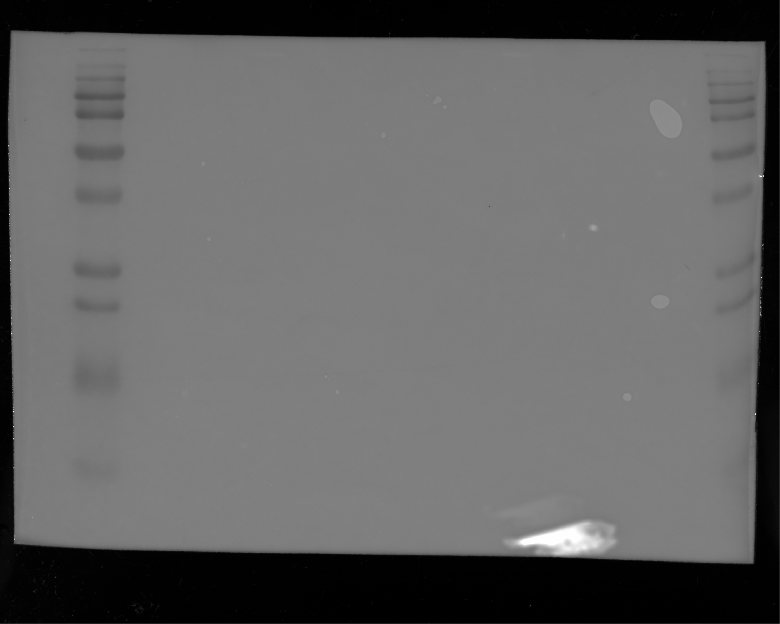 |

| **Target** | **kDa** | **Chemiluminescence** | **Colorimetric (Precision Plus Protein Standards #161-0374)** |
| --- | --- | --- | --- |
| Mcl-1 | 40 | 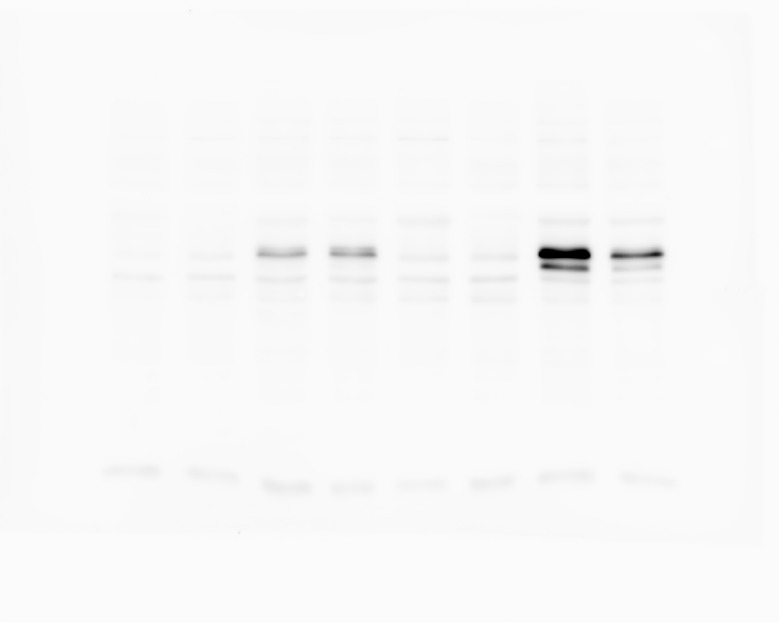 | 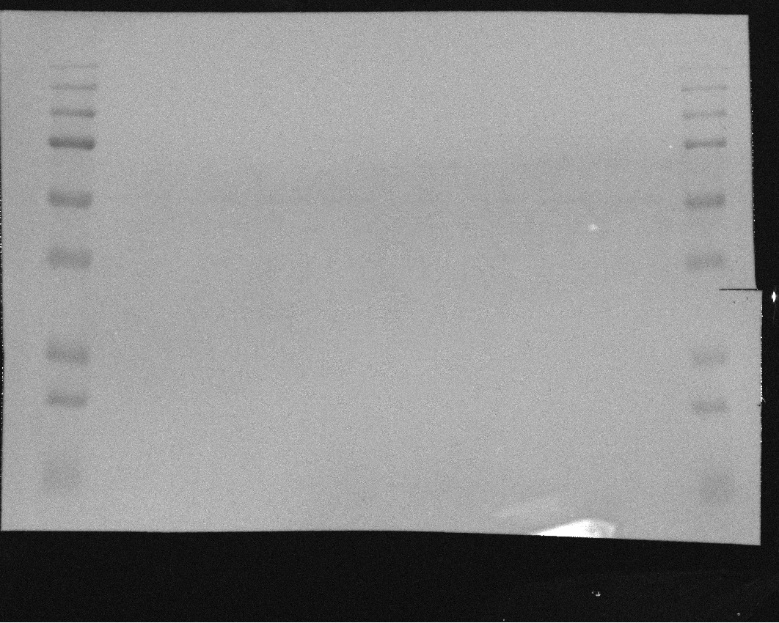 |
| α-tubulin to Mcl-1 | 50 | 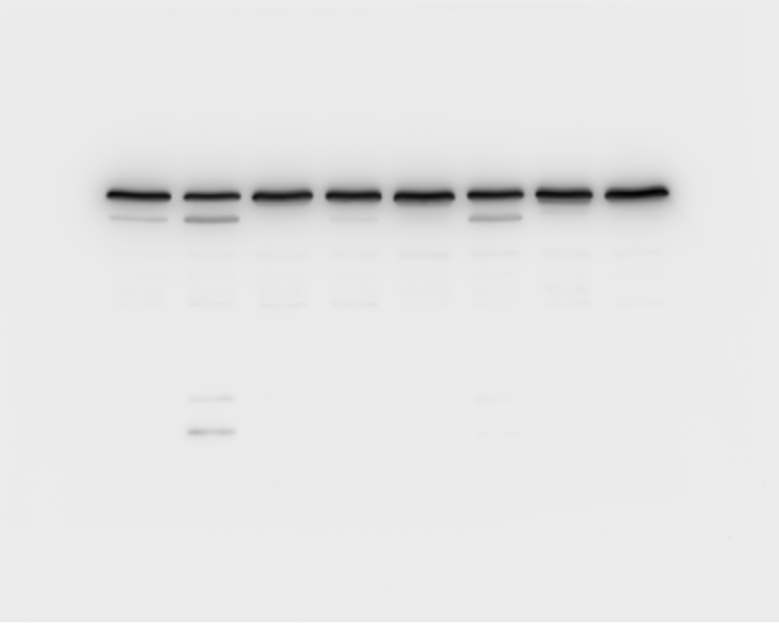 | 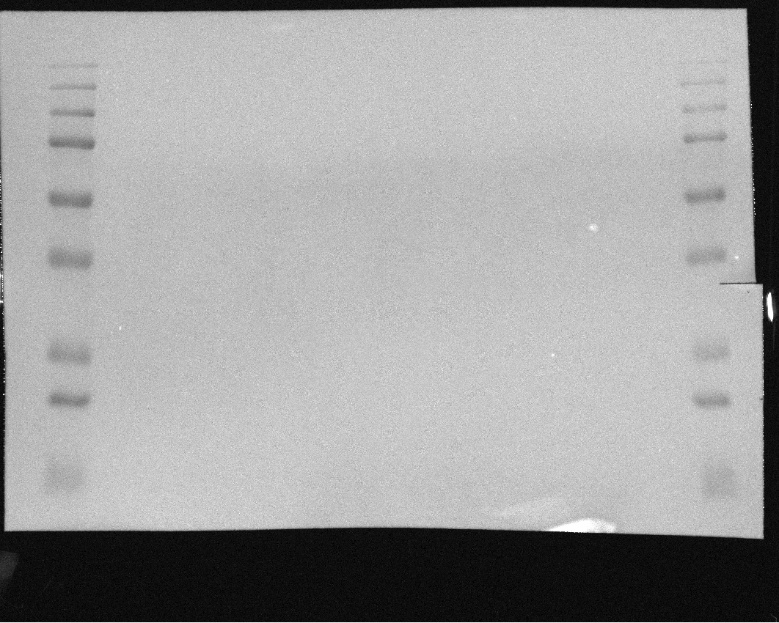 |

| **Target** | **kDa** | **Chemiluminescence** | **Colorimetric (Precision Plus Protein Standards #161-0374)** |
| --- | --- | --- | --- |
| Bcl-XL | 30 | 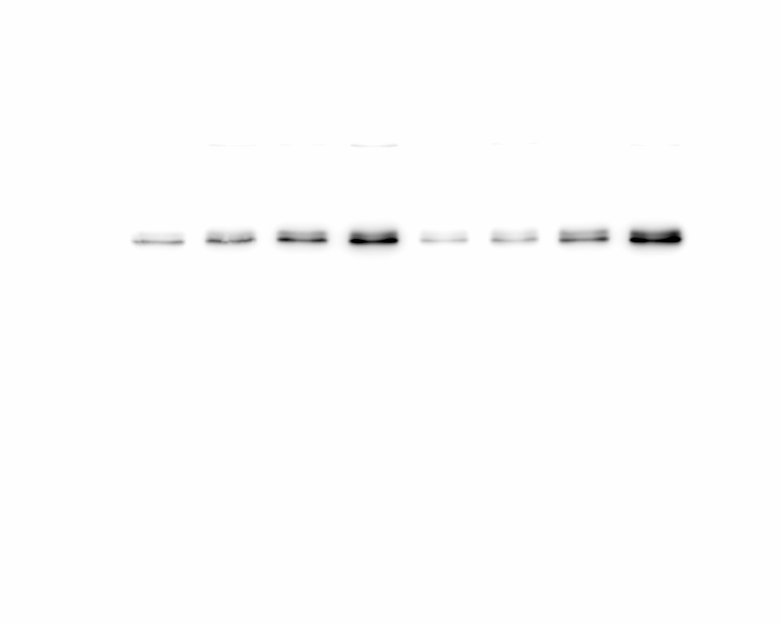 | 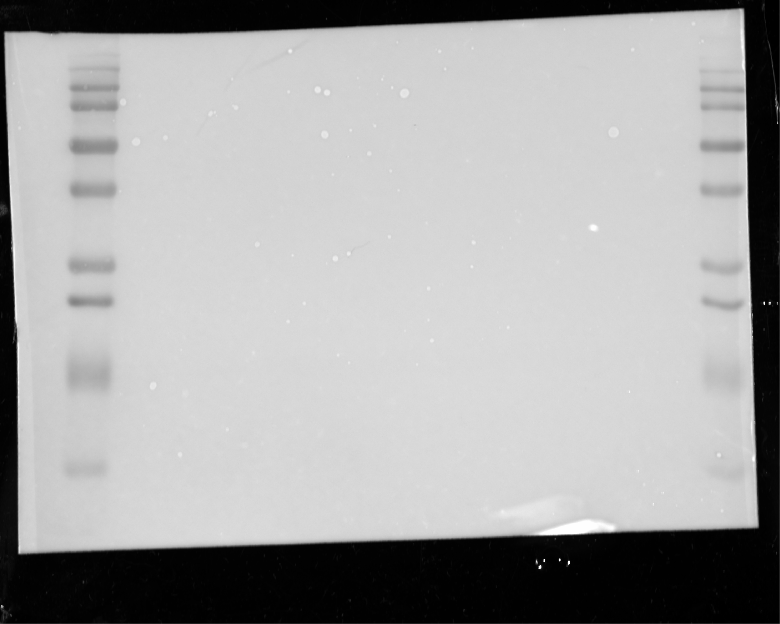 |
| α-tubulin to Bcl-XL | 50 | 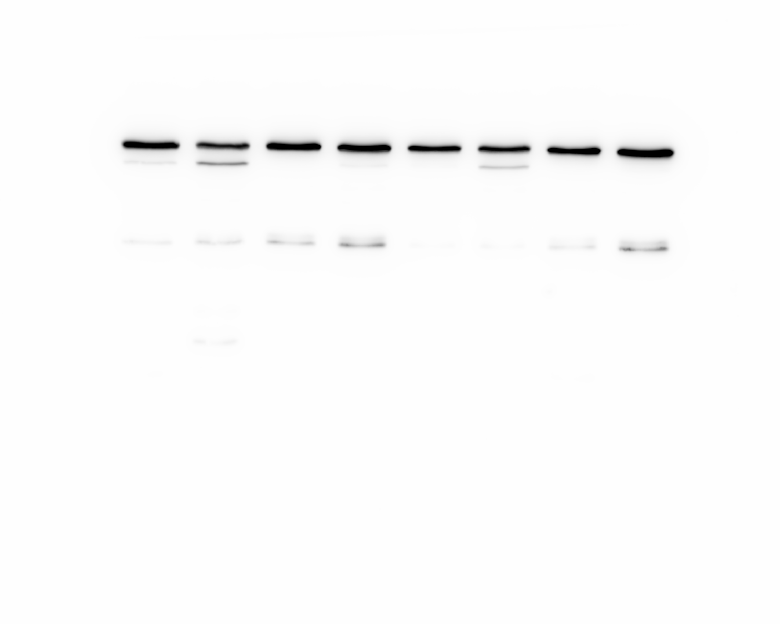 | 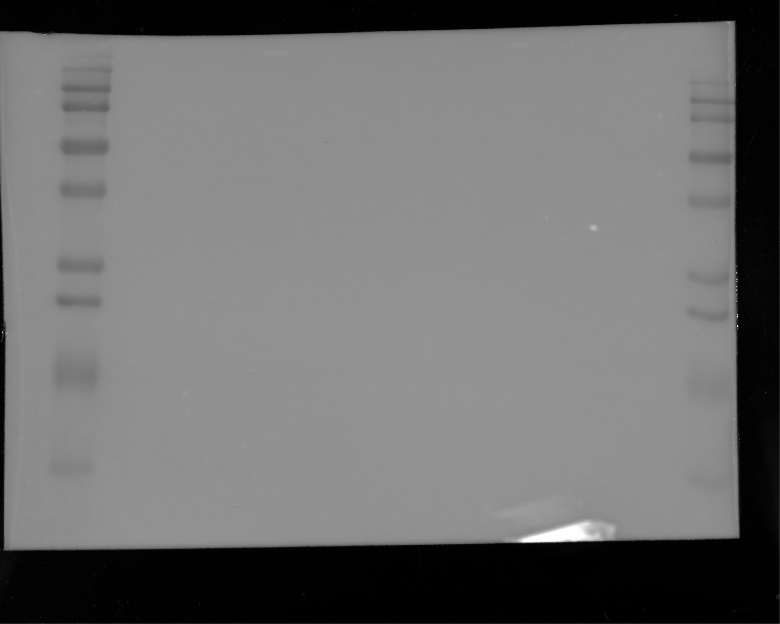 |

| **Target** | **kDa** | **Chemiluminescence** | **Colorimetric (Precision Plus Protein Standards #161-0374)** |
| --- | --- | --- | --- |
| BIM | 25, 15, 12 | 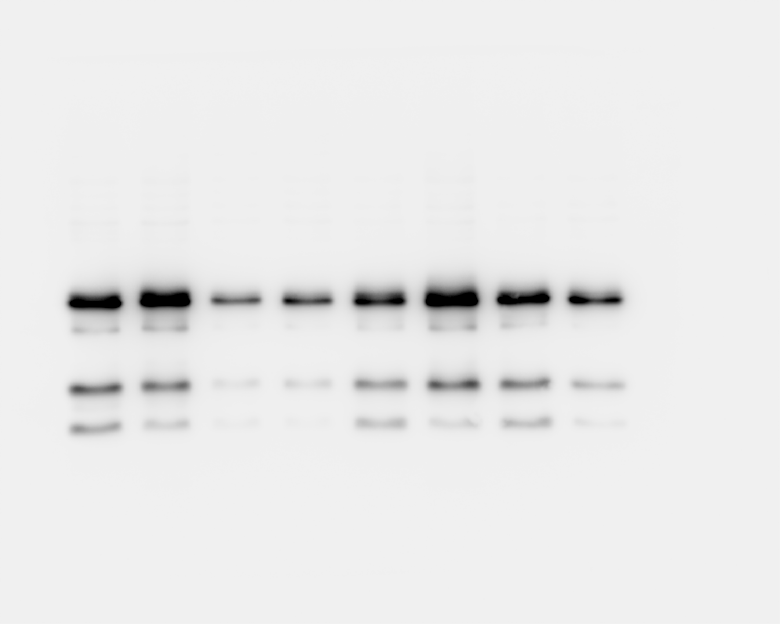 | 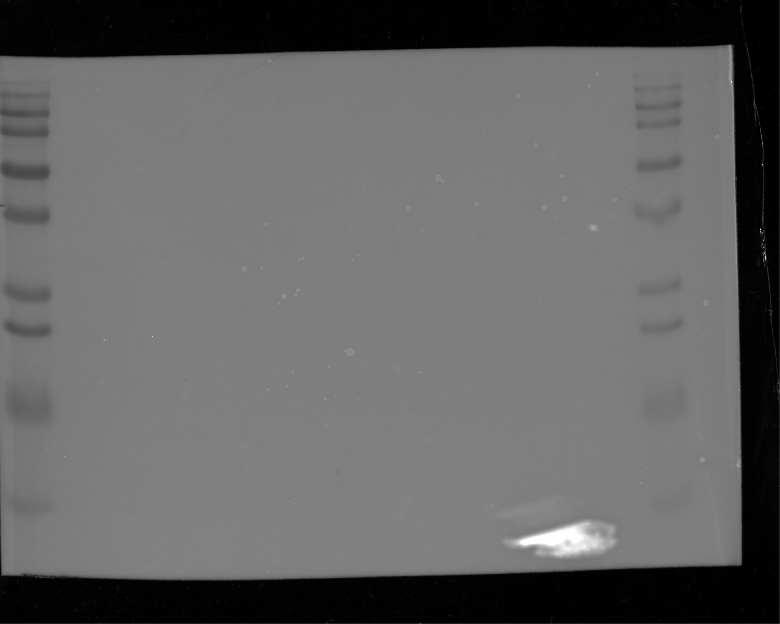 |
| α-tubulin to BIM | 50 | 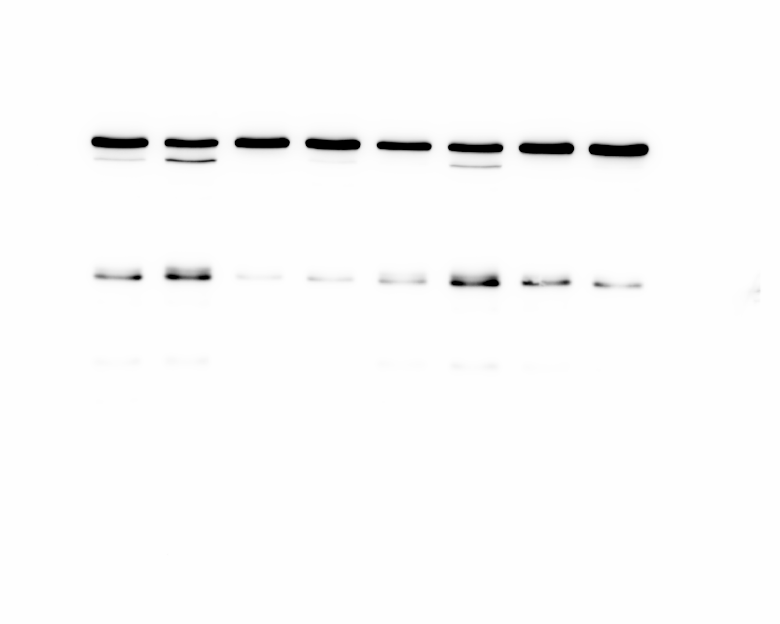 | 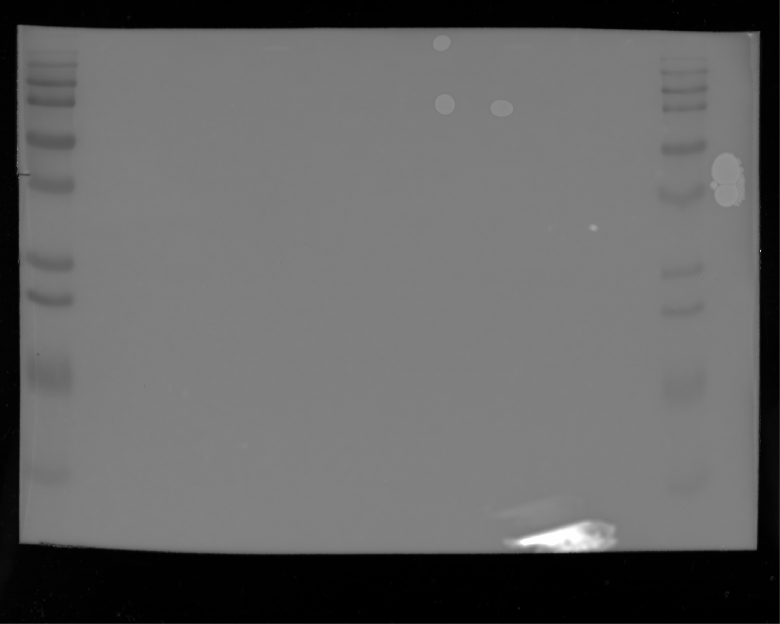 |

| **Target** | **kDa** | **Chemiluminescence** | **Colorimetric (Precision Plus Protein Standards #161-0374)** |
| --- | --- | --- | --- |
| NOXA | 10 | 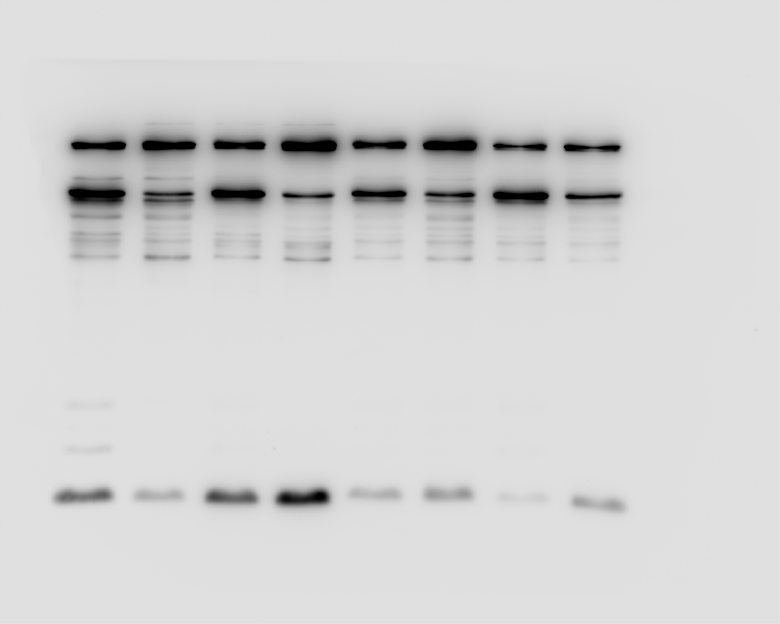 | 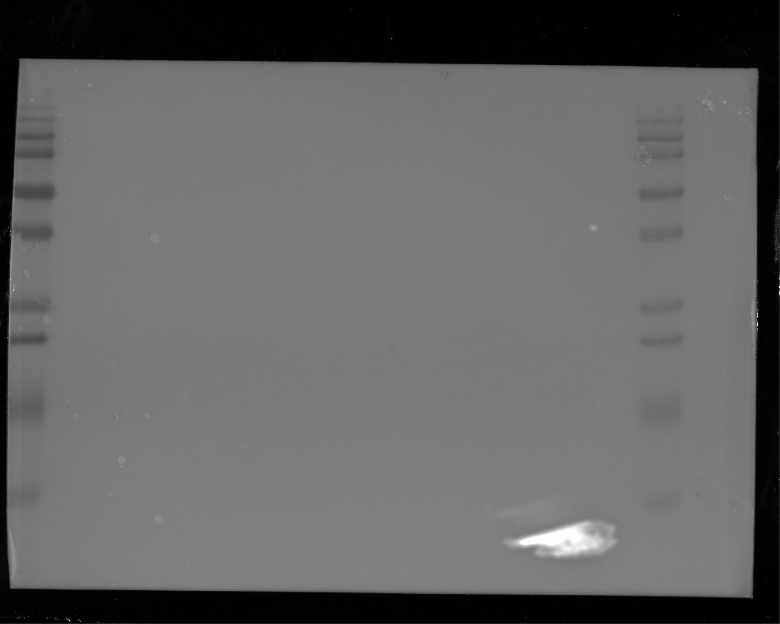 |
| α-tubulin to NOXA | 50 | 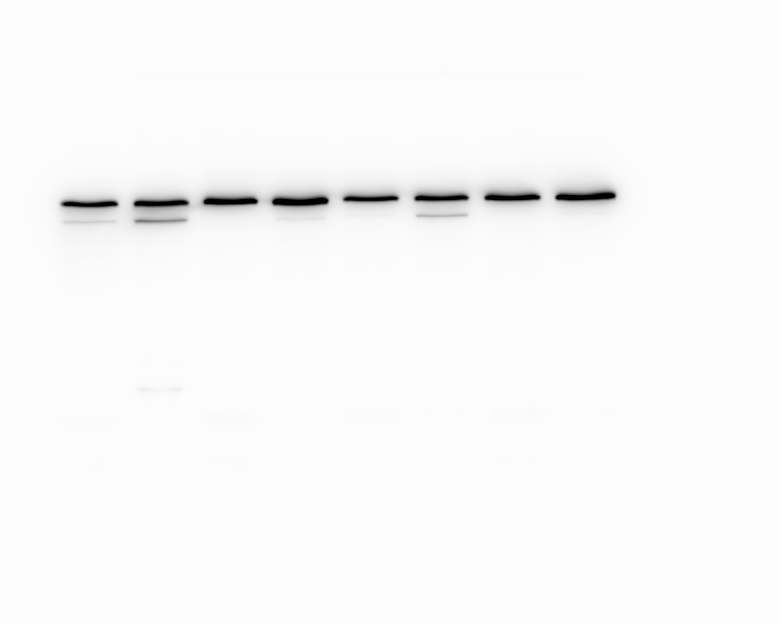 | 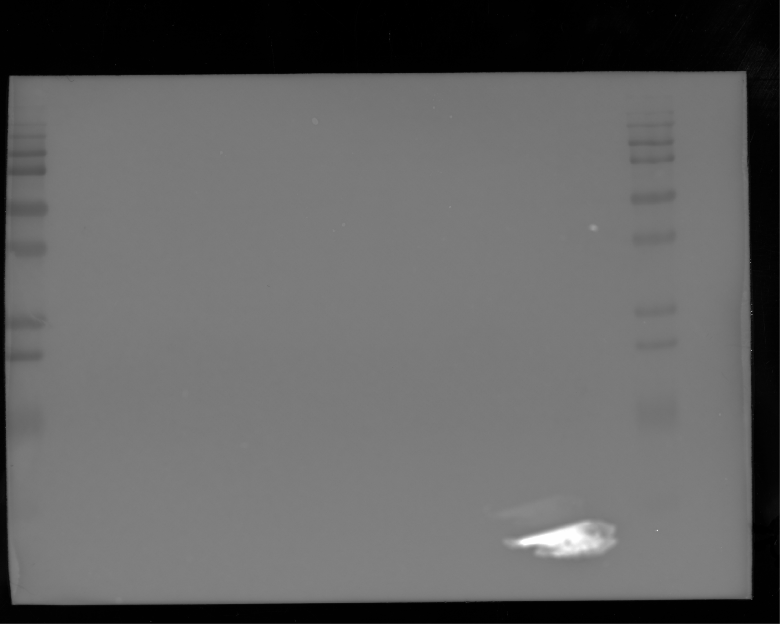 |

| **Target** | **kDa** | **Chemiluminescence** | **Colorimetric (Precision Plus Protein Standards #161-0374)** |
| --- | --- | --- | --- |
| Akt | 60 | 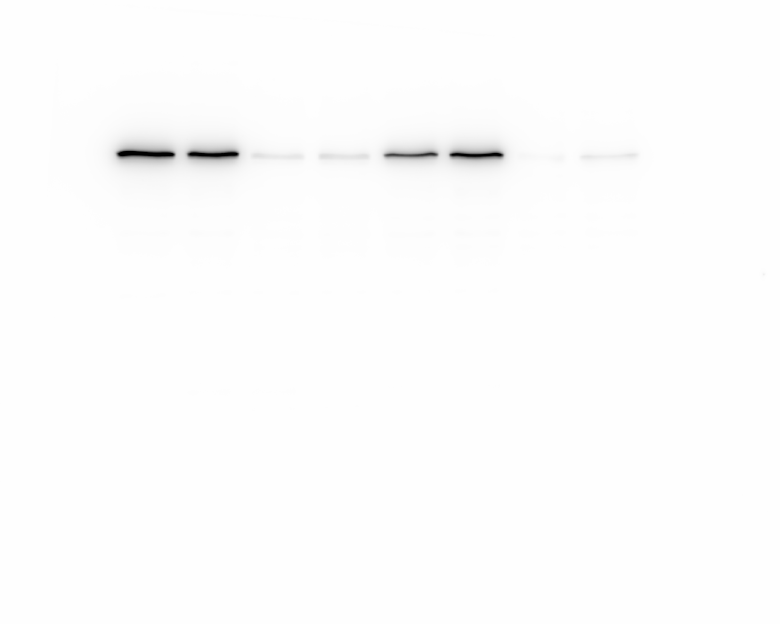 | 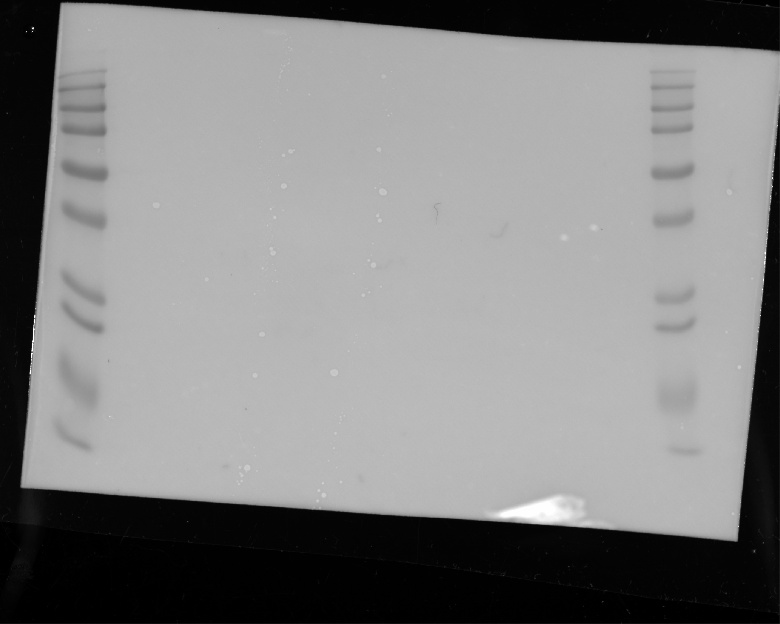 |
| beta-actin to Akt | 47 | 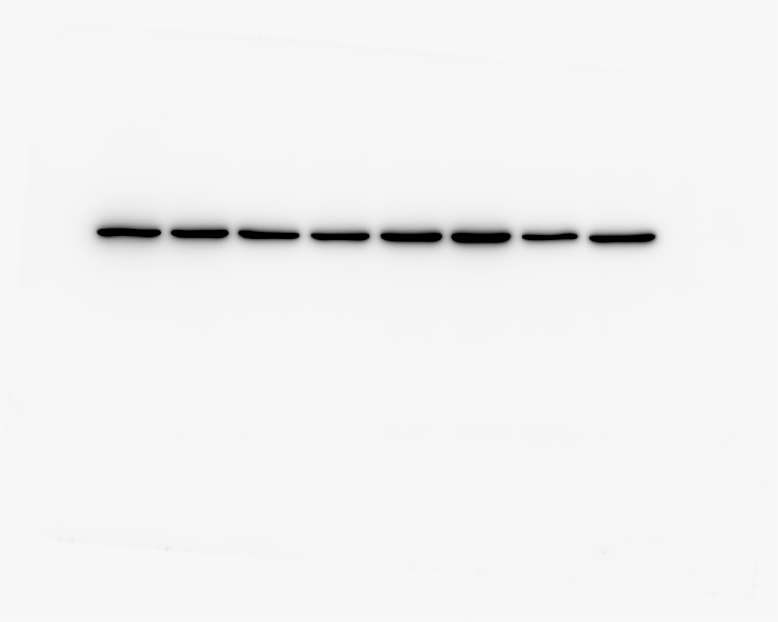 | 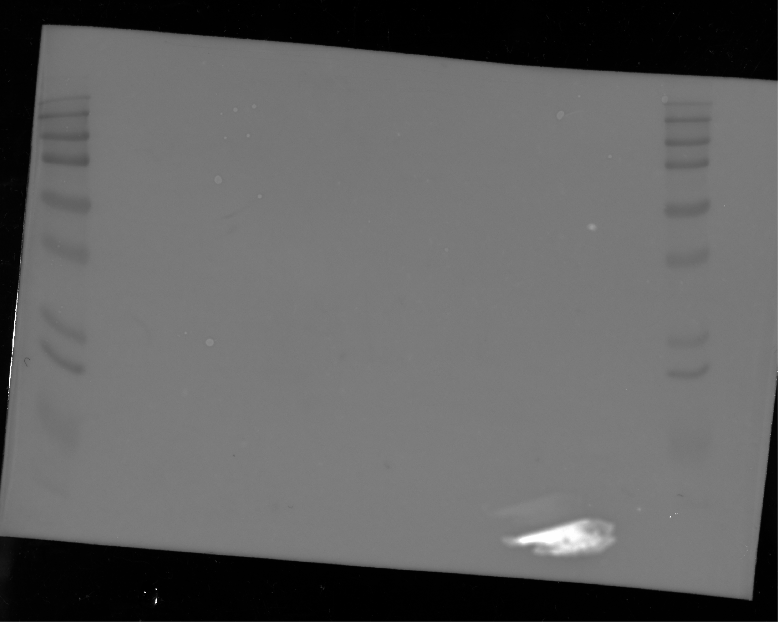 |

| **Target** | **kDa** | **Chemiluminescence** | **Colorimetric (Precision Plus Protein Standards #161-0374)** |
| --- | --- | --- | --- |
| pAkt | 60-65 | 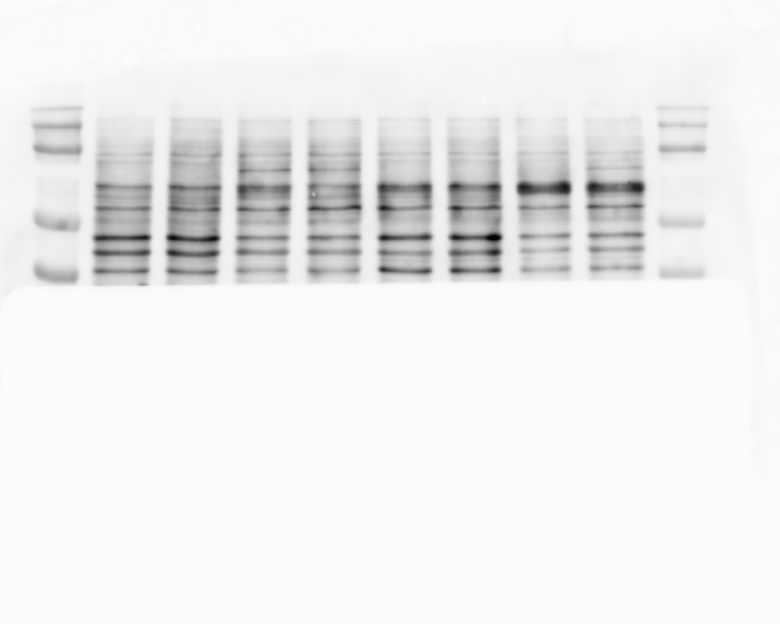 | 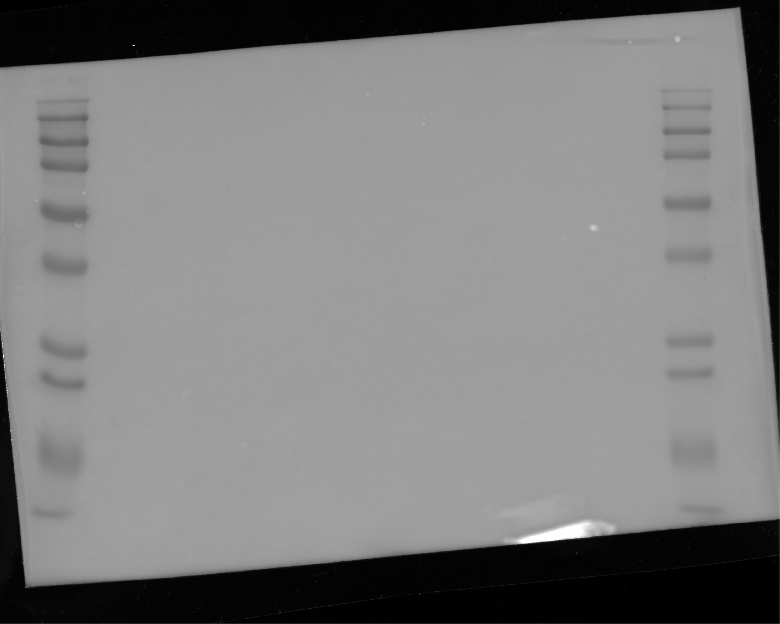 |
| beta-actin to pAkt | 47 | 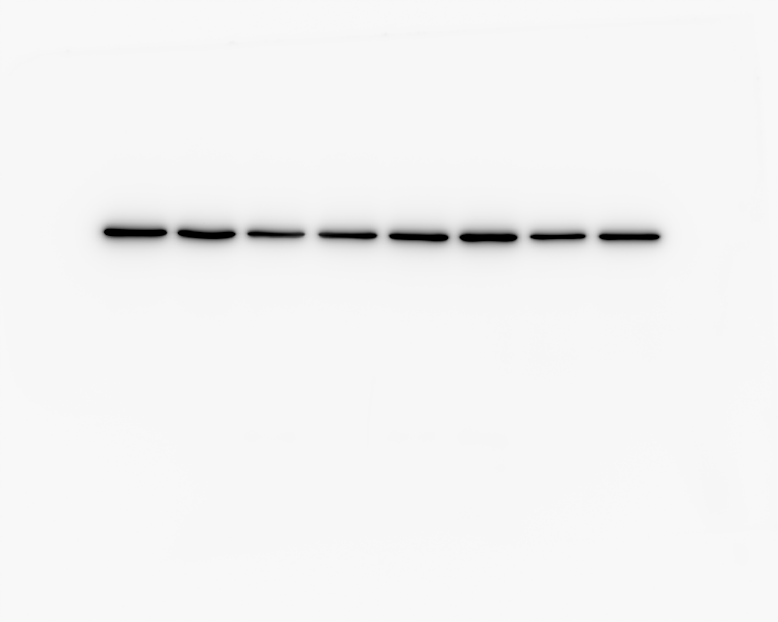 | 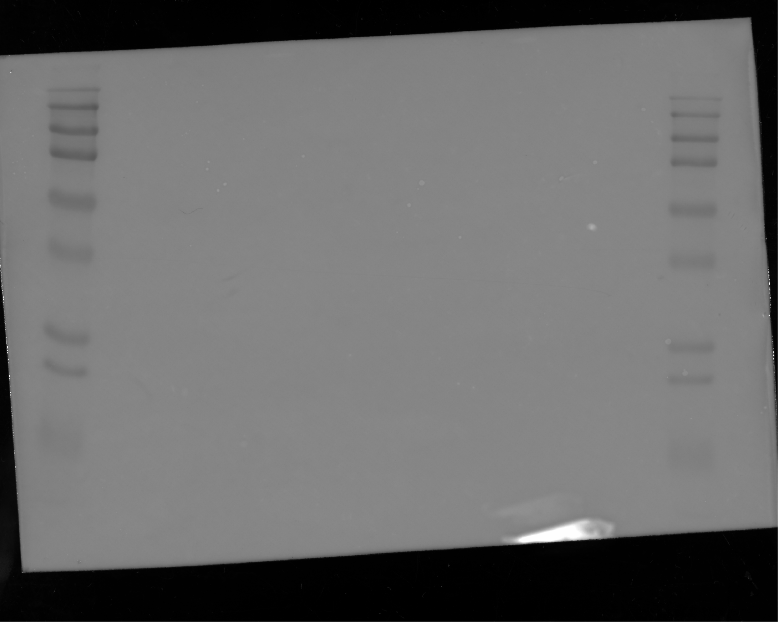 |
